# Ortholog of the polymerase theta helicase domain modulates DNA replication in *Trypanosoma cruzi*

**DOI:** 10.1038/s41598-019-39348-2

**Published:** 2019-02-27

**Authors:** Loyze P. de Lima, Simone G. Calderano, Marcelo S. da Silva, Christiane B. de Araujo, Elton J. R. Vasconcelos, Leo K. Iwai, Claudio A. Pereira, Stenio P. Fragoso, M. Carolina Elias

**Affiliations:** 1 Laboratorio Especial de Ciclo Celular, Instituto Butantan, São Paulo, Brazil; 2 Center of Toxins, Immune Response and Cell Signaling (CeTICS), Instituto Butantan, São Paulo, Brazil; 3 Laboratório de Parasitologia, Instituto Butantan, São Paulo, Brazil; 4 College of Veterinary Medicine, Western University of Health Sciences, Pomona, CA 91766 USA; 5 Laboratório Especial de Toxinologia Aplicada, Instituto Butantan, São Paulo, Brazil; 6 Laboratorio de Parasitología Molecular, Instituto de Investigaciones Médicas A. Lanari, Universidad de Buenos Aires - Consejo Nacional de Investigaciones Científicas y Técnicas (CONICET), Combatientes de Malvinas, (C1427ARO) Ciudad Autónoma de Buenos Aires, Buenos Aires, Argentina; 7 Instituto Carlos Chagas, Fiocruz-PR, Curitiba, Brazil

## Abstract

DNA polymerase theta (Polθ), a member of the DNA polymerase family A, exhibits a polymerase C-terminal domain, a central domain, and an N-terminal helicase domain. Polθ plays important roles in DNA repair via its polymerase domain, regulating genome integrity. In addition, in mammals, Polθ modulates origin firing timing and MCM helicase recruitment to chromatin. In contrast, as a model eukaryote, *Trypanosoma cruzi* exhibits two individual putative orthologs of Polθ in different genomic loci; one ortholog is homologous to the Polθ C-terminal polymerase domain, and the other is homologous to the Polθ helicase domain, called Polθ-polymerase and Polθ-helicase, respectively. A pull-down assay using the *T. cruzi* component of the prereplication complex Orc1/Cdc6 as bait captured Polθ-helicase from the nuclear extract. Orc1/Cdc6 and Polθ-helicase directly interacted, and Polθ-helicase presented DNA unwinding and ATPase activities. A *T. cruzi* strain overexpressing the Polθ-helicase domain exhibited a significantly decreased amount of DNA-bound MCM7 and impaired replication origin firing. Taken together, these data suggest that Polθ-helicase modulates DNA replication by directly interacting with Orc1/Cdc6, which reduces the binding of MCM7 to DNA and thereby impairs the firing of replication origins.

## Introduction

DNA polymerase theta (Polθ) is an A family polymerase that functions in genomic maintenance; Polθ has homology to *E. coli* Pol I^1^ and is widespread in multicellular eukaryotes but not in fungi^2,3^. Polθ is involved in the repair of double-stranded breaks (DSBs) in DNA via microhomology-mediated end joining (MMEJ), an alternative error-prone repair mechanism for DSBs. In this process, Polθ utilizes short microhomologies to join the two DNA strands^4^. The role of Polθ in MMEJ has already been demonstrated in *Drosophila*^5^, *C. elegans*^6^, zebrafish^7^, and mice^8^. In addition to DNA repair, Polθ also plays a role in DNA replication, which beings at the G1 phase of the cell cycle with assembly of the prereplication complex; heterohexamer origin recognition complex (ORC), composed of Orc1 to Orc6, binds to DNA regions, licensing them as replication origins. Once bound to DNA, ORC recruits Cdc6, and together, ORC and Cdc6 recruit Cdt1 and the minichromosome maintenance (MCM) complex, which is composed of six subunits (MCM2 to MCM7) and has helicase activity fundamental for DNA replication. Once cells reach the S phase, other regulatory and enzymatic players are recruited to DNA origins, and DNA replication is then established^9^. In mammalian cells, Polθ is immunoprecipitated with Orc2 and Orc4, strongly suggesting that Polθ is part of the prereplication complex, although the exact mechanisms of interaction are unknown. The interaction of Polθ with the prereplication complex inhibits the recruitment of MCM to the origin, thereby modulating the origin firing timing during S phase^10^.

Polθ exhibits a C-terminal DNA polymerase domain and an N-terminal helicase-like domain, which has DNA-dependent ATPase activity^11^; a long central domain of unknown function separates these domains. The DNA polymerase domain is fundamental for the action of Polθ in MMEJ. *In vitro*, the Polθ polymerase domain can join DNA ends with microhomology and mediate the alignment of internal and terminal microhomologous sequences^12^. Moreover, this domain is essential for interstrand DNA crosslink (ICL) repair^11^. Little is known about the importance of the Polθ helicase domain in DNA repair, but mutation of the helicase domain was recently shown to impair efficient DNA break joining^13^.

Trypanosomatids are a group of protozoan parasites that includes human pathogens of substantial medical relevance, such as *Trypanosoma cruzi* (etiological agent of Chagas disease), *Trypanosoma brucei* (etiological agent of African sleeping sickness), and *Leishmania spp*. (etiological agent of distinct forms of leishmaniasis). These parasites belong to the Excavata supergroup, which diverged early during eukaryotic evolution, and have drawn attention as models for genetic, evolutionary and comparative studies. A putative ortholog of DNA Polθ (LiPolθ) protects *Leishmania infantum* against oxidative damage and thus exhibits a translesion synthesis polymerase activity. LiPolθ shares homology with the C-terminal polymerase of Polθ but lacks the N-terminal helicase domain^14^. Because we found two orthologs of Polθ in *T. cruzi*, one containing the DNA polymerase domain and the other containing the helicase domain, we asked whether Polθ could interact with ORC in *T. cruzi*. In trypanosomatids, ORC is highly divergent from model eukaryotes^15^, but we and others have previously shown that Orc1/Cdc6 is an ORC component that participates in DNA replication^16,17^. Here, a *T. cruzi* Orc1/Cdc6 pull-down was able to capture the putative ortholog of the N-terminal region of Polθ containing the helicase and ATPase motifs. We then expressed and purified the recombinant Polθ-helicase and demonstrated that this protein exhibits both ATPase and helicase activities. The recombinant Polθ-helicase directly interacts with the recombinant TcOrc1/Cdc6 and is bound to DNA throughout the cell cycle. Overexpression of Polθ-helicase reduces the level of MCM helicase on DNA and impairs the firing of replication origins. Our data show that without the polymerase domain, *T. cruzi* Polθ-helicase directly interacts with Orc1/Cdc6 and functions as a limiting factor that modulates the binding of MCM to DNA, thus downregulating replication.

## Results

### Putative *T. cruzi* Polθ polymerase and helicase domains

**Figure 1:**
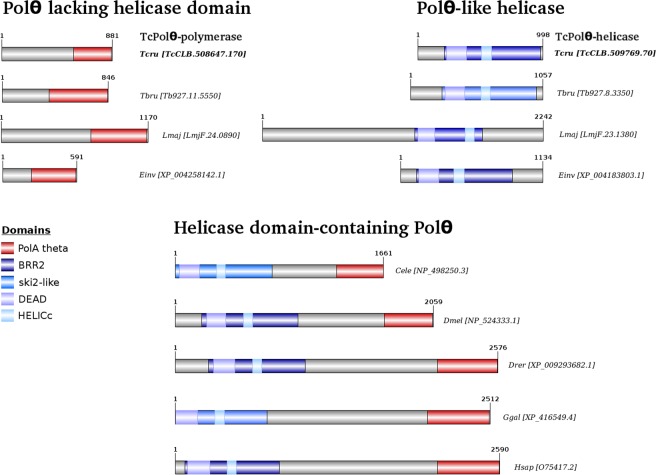
Schematic representation of DNA polymerase A theta protein in several eukaryotes of different evolutionary clades. The primitive protozoan parasites *Trypanosoma cruzi* (Tcru), *T. brucei* (Tbru), *Leishmania major* (Lmaj), and Entamoeba *invadens* (Einv) (the latter being from a distinct phylum compared to the others), exhibit two independent genes encoding domains that might be associated with Polθ activity, replicative superfamily II helicase (BRR2, or ski2-like helicase), which comprises two shorter domains involved in the helicase function (DEAD/DEAH box and HELICc), and the DNA PolA theta domain itself. On the other hand, multicellular organisms (*Caenorhabditis elegans* (Cele), *Drosophila melanogaster* (Dmel), *Danio rerio* (Drer), *Gallus gallus* (Ggal) and *Homo sapiens* (Hsap)) have these same domains in one single Polθ gene/protein. The identities and percent similarities of all the depicted proteins compared to *T. cruzi* proteins are shown in Supplementary Table 1.

The Polθ amino acid structure is conserved among metazoans, exhibiting a C-terminal DNA polymerization core domain, essential for the action of Polθ during DNA repair, and an N-terminal helicase domain, which exhibits DNA-dependent ATPase activity (Fig. 1). To confirm the presence of and establish the position of the domains and motifs in Polθ from *T. cruzi*, we carried out an *in silico* analysis with the access codes provided by BLAST analysis^18^ using the two *T. cruzi* Polθ sequences as the query (Supplementary Table 1). Our *in silico* analysis confirmed the identities of two independent *T. cruzi* genes (TcCLB.508647.170 and TcCLB.509769.70), which separately encode helicase and polymerase domains, and compared their similarities to genes functionally annotated as Polθ in higher eukaryotes (Fig. 1 and Supplementary Table 1). The helicase domain is named replicative superfamily II helicase (BRR2), or ski2-like helicase, and comprises two shorter domains involved in helicase function (DEAD/DEAH box and HELICc), while the polymerase domain is named the DNA PolA θ domain. *T. brucei* and *L. major* orthologs are presented in Fig. 1 and Supplementary Table 1 along with those of *T. cruzi*. These trypanosomatids also present PolQ domains in two distinct open reading frames (ORFs). Two homologs of Polθ also exist in mammals, DNA polymerase nu (POLN) and helicase PolQ-like (HELQ)^3,19^, and we found BLASTP hits against HELQ (using *T. cruzi* Polθ-helicase as the query) and POLN (using *T. cruzi* Polθ-polymerase as the query) (Supplementary Table 1). Therefore, *T. cruzi* Polθ-helicase is feasibly a HELQ homolog, while *T. cruzi* Polθ-polymerase is feasibly a POLN homolog.

### The Polθ-helicase domain directly interacts with the ORC component Orc1/Cdc6

**Figure 2:**
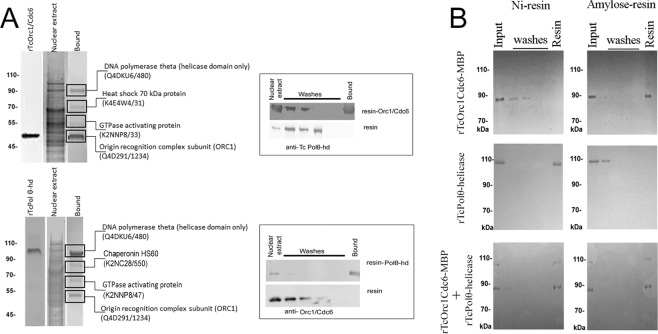
rTcOrc1/Cdc6 and rPolθ-helicase are directly associated. (**A**) rTcOrc1/Cdc6 (top panel) or rPolθ-helicase (bottom panel) was used as bait to pull down epimastigote nuclear proteins. Proteins bound to recombinant protein were excised from the gel and analyzed by mass spectrometry. Proteins that also precipitated in the absence of recombinant protein were excluded. Proteins bound to recombinant protein or only to resin were subjected to western blot analysis using anti-TcPolθ helicase or anti-Orc1/Cdc6 (inserted boxes). (**B**) rTcOrc1/Cdc6-MBP (top panel), his-rPolθ-helicase (middle panel) or both proteins (bottom panel) were incubated with Ni resin (left panel) or amylose resin (right panel). The elution lane shows proteins that were captured by each resin.

Because we found that one of the Polθ orthologs in *T. cruzi* contains the DNA polymerase domain while the other contains the helicase domain, we evaluated whether either of the Polθ domains could interact with ORC in *T. cruzi* using *T. cruzi* recombinant Orc1/Cdc6 (rTcOrc1Cdc6), purified as previously described^16^, as bait in a pull-down assay. Proteins captured from *T. cruzi* epimastigote nuclear extracts using rOrc1/Cdc6 and those captured using only resin as a control were subjected to SDS-PAGE and analyzed by mass spectrometry. After excluding the proteins captured by resin (unspecific binding), ORC1, the protein we called Orc1/Cdc6, and the Polθ helicase domain were retained (Fig. 2A, top panel). To confirm this result, we subjected proteins captured by Orc1/Cdc6 or resin to western blot analysis using anti-Tc Polθ helicase (Fig. 2A, superior box) and revealed that Polθ helicase was indeed captured by Orc1/Cdc6 but not by resin. To demonstrate that Orc1/Cdc6 and the Polθ helicase domain are part of the same complex, we inverted the strategy. We cloned, expressed and purified the Polθ helicase domain (Fig. S1) and used the recombinant rPolθ helicase domain as bait in a pull-down assay. Proteins captured from the *T. cruzi* epimastigote nuclear extract using the rPolθ helicase domain and those captured using only the resin as a control were subjected to SDS-PAGE and analyzed by mass spectrometry. After excluding proteins captured by resin (unspecific binding), Polθ-helicase and Orc1/Cdc6 were retained (Fig. 2A, bottom panel). We also subjected proteins captured by Polθ helicase or resin to western blot analysis using anti-Tc Orc1/Cdc6 (Fig. 2A, inferior box), revealing that Orc1/Cdc6 was indeed captured by Polθ helicase but not by resin. Because our data suggested that Orc1/Cdc6 and Polθ-helicase are part of the same complex, we next evaluated whether they could interact with each other. We first purified rTcOrc1/Cdc6 fused to maltose binding protein (rTcOrc1/Cdc6-MBP), as described previously^16^, and showed that this recombinant protein was captured by amylose resin but not by Ni resin (Fig. 2B, top panel). Then, we showed that rPolθ-helicase fused to a histidine tag (rPolθ-helicase-his) was captured by Ni resin but not by amylose resin (Fig. 2B, middle panel). Finally, we mixed rPolθ-helicase-his and rTcOrc1/Cdc6-MBP *in vitro* and found that rPolθ-helicase-his was captured by amylose resin, indicating that it was captured by rTcOrc1/Cdc6-MBP. In the opposite strategy, we found that rTcOrc1/Cdc6-MBP bound to Ni resin, indicating that it was captured by rPolθ-helicase-his (Fig. 2B, bottom panel). Together, these results show that Polθ-helicase directly interacts with Orc1/Cdc6.

### rPolθ-helicase presents ATPase and helicase activities

**Figure 3:**
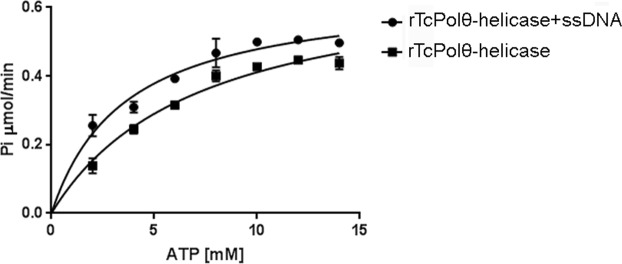
rPolθ-helicase presents ATPase activity. rPolθ-helicase was maintained in the presence of crescent concentrations of ATP with or without 2.5 ng of denatured salmon sperm DNA (ssDNA). The amount of inorganic phosphate released was determined.

**Figure 4:**
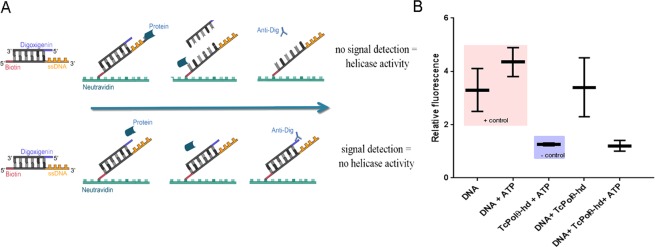
rPolθ-helicase presents ATP-dependent helicase activity. (**A**) Schematic representation of the helicase assay. Partial double-stranded DNA was composed of one oligonucleotide labeled with digoxigenin and another labeled with biotin; the dsDNA contained a 3′ tail to initiate unwinding. DNA was bound to a NeutrAvidin-sensitized plate via its biotinylated strand. After incubation with recombinant protein, the helicase activity was measured by analyzing the presence of strands labeled with digoxigenin using an anti-digoxigenin antibody. The absence of a signal indicated helicase activity. (**B**) The helicase assay was performed with only DNA or DNA and ATP (pink box – negative control: no helicase activity – detection of digoxigenin), with no DNA (purple box – no detection of digoxigenin), and in the presence of DNA and rPolθ-helicase in the presence or absence of ATP.

Because mammalian Polθ exhibits an N-terminal helicase domain with ATPase and helicase activities^19–21^, we first evaluated whether rPolθ-helicase had the ability to hydrolyze ATP in the presence or absence of single-stranded DNA (ssDNA). rPolθ-helicase was incubated with increasing amounts of ATP, and the release of Pi was quantified, revealing that both activities followed a Michaelis-Menten kinetic model (R^2^ = 0.9646 for activity in the absence of DNA and R^2^ = 0.9259 for activity in the presence of ssDNA; both curves were adjusted to the theoretical Michaelis-Menten hyperbolic function). Like mammalian Polθ, rPolθ-helicase had a higher affinity for ATP in the presence of ssDNA (K_M_ = 3.588 ± 0.5845) than in the absence of ssDNA (K_M_ = 7.168 ± 1.133), and the difference was statistically significant (p value < 0.05) (Fig. 3). We then tested whether rPolθ-helicase actually exhibited helicase activity using a recently described method. In this assay, we used partially double-stranded DNA (dsDNA); one strand was labeled with biotin, and the other was labeled with digoxigenin. DNA was then attached to a plate sensitized with streptavidin, which binds to biotin strands. After incubating DNA with rPolθ-helicase in the presence or absence of ATP, the digoxigenin-labeled strand was evaluated using an anti-digoxigenin antibody; detection of the digoxigenin-labeled strand indicated no helicase activity because dsDNA was maintained, while no detection of a digoxigenin-labeled strand indicated helicase activity (Fig. 4A). The ssDNA extension can be utilized to establish initial contact between helicase and DNA^22^, and we herein used a dsDNA containing a ssDNA 3′ tail in this assay (Fig. 4A), revealing that rPolθ-helicase exhibits ATP-dependent helicase activity and is capable of unwinding DNA in the 3′-5′ direction (Fig. 4B).

### Polθ-helicase is localized in the nucleus and interacts with DNA

**Figure 5:**
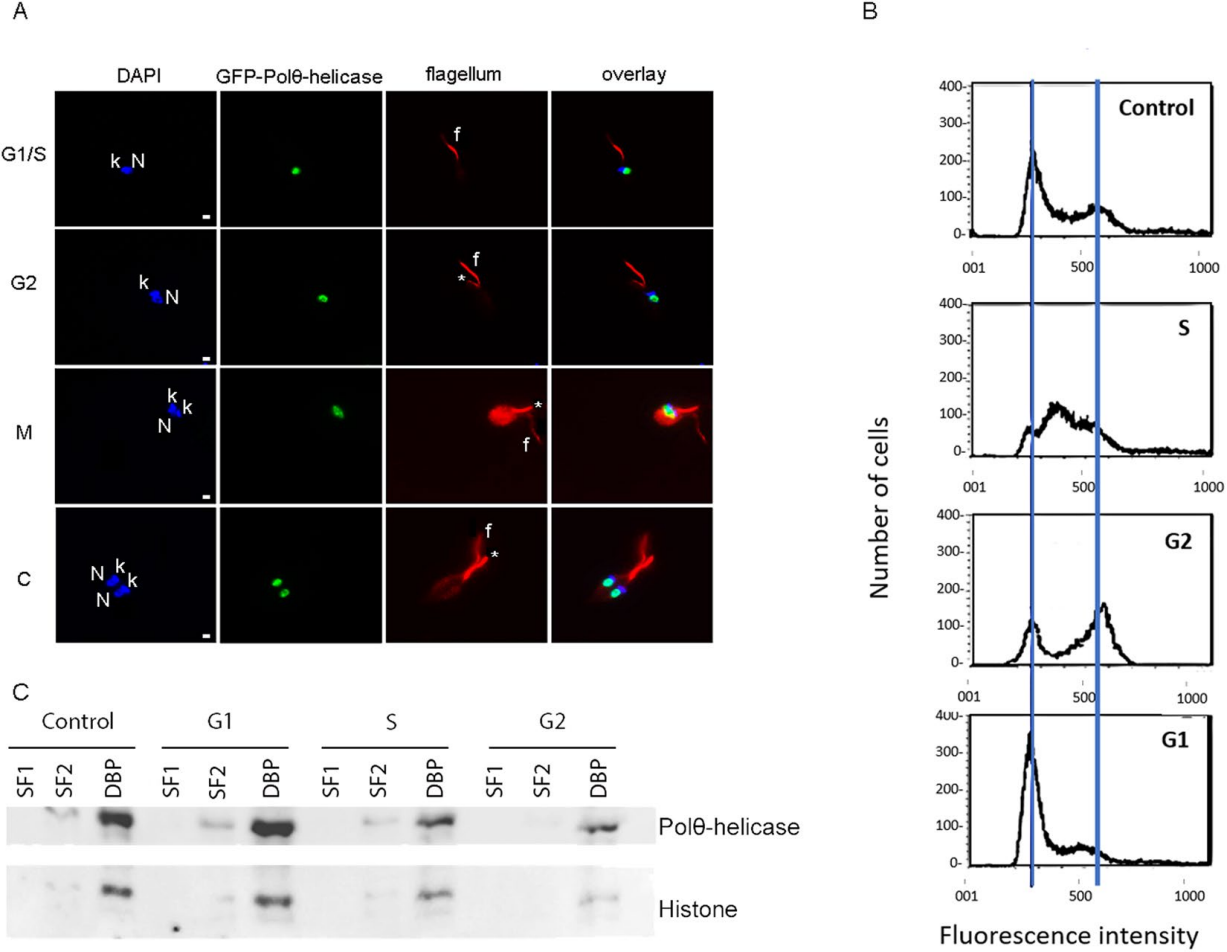
Polθ-helicase is bound to DNA throughout the entire cell cycle. (**A**) Epimastigote cells overexpressing GFP-Polθ-helicase were fixed, permeabilized, incubated with mAbAC, which recognizes flagellum (red), and stained with DAPI (blue). N – nucleus, k – kinetoplast, f – flagellum and * – new flagellum. Bars represent 2 μM. (**B**) Epimastigote cells were treated with HU for 24 h, washed and maintained in culture for 6 h (S), 18 h (G2), and 24 h (G1). “Control” indicates cells that were not treated. Cells were stained with propidium iodide and analyzed according to their DNA content. Blue lines indicate the peak of the fluorescence intensity of G1/S cells (left line) and G2/M cells (right line). (**C**) The samples analyzed in (**B**) were subjected to cell fractionation. In this assay, cells were lysed, and after centrifugation, the supernatants were saved as soluble fraction 1 (SF1). Again, pellets were incubated with lysis buffer and centrifuged, and the supernatants were saved as soluble fraction 2 (SF2). Finally, pellets were treated with DNase to obtain DNA-bound proteins (DBPs) and then centrifuged; the supernatants were saved as DBPs. Samples were subjected to western blotting using anti-Polθ-helicase and anti-histone H3 as a control in DBP fractions.

Because Polθ-helicase interacts with Orc1/Cdc6, which is located in the nucleus and bound to DNA throughout the entire cell cycle^16^, we examined whether Polθ-helicase was localized in the nucleus and associated with DNA during the *T. cruzi* epimastigote cell cycle. We generated an epimastigote lineage overexpressing Polθ-helicase fused to GFP via its N-terminal region (GFP-Polθ-helicase), and the nuclear GFP signal was detected in 100% of the cells analyzed. These data show that Polθ-helicase is located in the nucleus throughout the entire cell cycle. To corroborate these data, we analyzed the number of nuclei, kinetoplasts and flagella in cells containing the nuclear GFP signal, as this is an efficient way to determine the epimastigote cell cycle stage^23^. We observed G1/S cells (containing one nucleus, one kinetoplast and one flagellum), G2 cells (containing one nucleus, one kinetoplast and two flagella), mitotic cells (containing one nucleus, two kinetoplasts and two flagella) and cells at cytokinesis (two nuclei, two kinetoplasts and two flagella) that expressed GFP-Polθ-helicase in the nucleus (Fig. 5A). We then evaluated whether Polθ-helicase was bound to DNA throughout the entire cell cycle by first synchronizing cells with hydroxyurea (HU)^24^. After synchronization, HU was removed, and cells were maintained in culture for 6 h to obtain cells at the S phase, for 18 h to obtain cells at the G2 phase, and for 24 h to obtain cells that had went through mitosis and cytokinesis, reaching the G1 stage (Fig. 5B). Then, these cells were treated with lysis buffer to extract soluble proteins, and the remaining pellets were then treated with DNase to extract DNA-binding proteins (DBPs). Both fractions [soluble fraction (SF) and DBPs] from each sample were analyzed by western blot using an anti-Polθ-helicase antibody and anti-histone H3 as a control for the DBP fraction. Polθ-helicase was clearly associated with DNA during and outside of the S phase (Fig. 5C).

### Overexpression of Polθ-helicase impairs DNA replication

**Figure 6:**
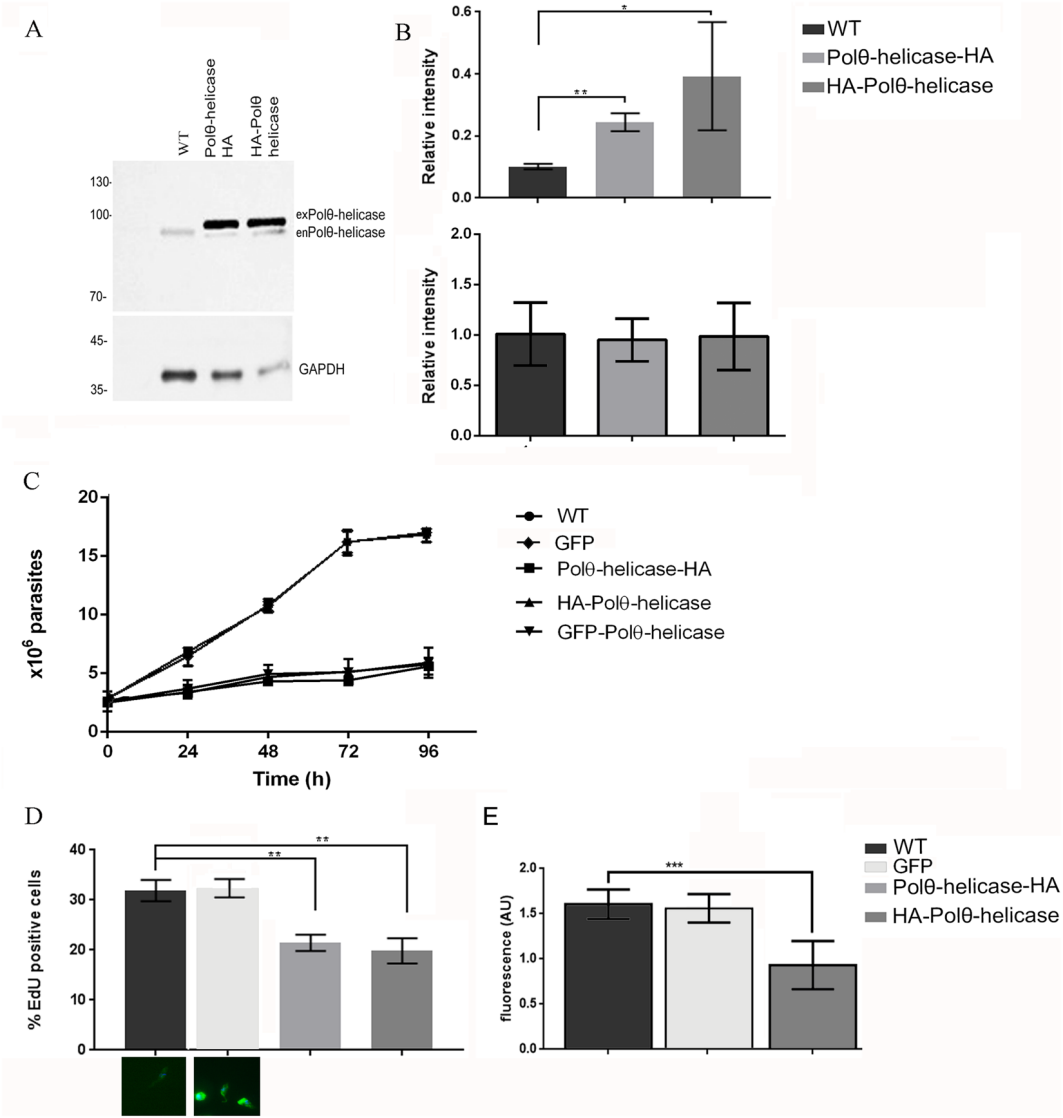
Polθ-helicase overexpression impairs DNA replication. (**A**) Proteic extract of control cells (WT) and cells overexpressing Polθ-helicase fused to HA via its C-terminus (Polθ-helicase-HA) or N-terminus (HA- Polθ-helicase) were subjected to western blotting using an anti-Polθ-helicase antibody or anti-GAPDH as the loading control. Endogenous Polθ-helicase is enPolθ-helicase, and exogenous protein is exPolθ-helicase. (**B**) The graph shows the quantification of both bands (Polθ-helicase with or without HA) presented in (**A**). The top graph is the quantification of exogenous Polθ-helicase, and the bottom graph is the quantification of endogenous Polθ-helicase. Values are expressed as the median and standard deviation of three independent experiments. (**C**) Growth cultures of control cells (WT) and cells overexpressing GFP, Polθ-helicase-HA or HA-Polθ-helicase. (**D**) The graph shows the percentage of cells that incorporated EdU. Values are expressed as the median and standard deviation of three independent experiments. One hundred cells were analyzed in each replicate. (**E**) The fluorescence intensity of cells labeled with EdU was measured using ImageJ. Values are expressed as the median and standard deviation of three independent experiments. One hundred cells were analyzed in each replicate. Statistical analyses were performed using Student’s t-test, *Indicates p < 0.05, **Indicates p < 0.01 and ***p < 0.001. The panels below the graph show GFP fluorescence (green channel) in GFP-overexpressing cells.

The association of Polθ-helicase with Orc1/Cdc6 strongly suggests the involvement of Polθ-helicase in nuclear DNA replication. To examine this possibility, we overexpressed Polθ-helicase fused to a hemagglutinin-antigen (HA) tag at its C-terminal (Polθ-helicase-HA) or N-terminal (HA-Polθ-helicase) region. We found Polθ-helicase expression to be increased by 2.6- and 3.8-fold in these overexpression lines compared to that in the control (Fig. 6A and B), and growth curves of both lineages were impaired (Fig. 6C). We also constructed a growth curve for GFP-Polθ-helicase and found very similar results (Fig. 6C). We then tested the abilities of the lineages overexpressing Polθ-helicase to replicate DNA. Control cells and cells overexpressing Polθ-helicase-HA and HA-Polθ-helicase were subjected to the 5-ethynyl-2′-deoxyuridine (EdU) incorporation assay, which revealed that cultures overexpressing Polθ-helicase exhibited a reduced number of replicating cells (Fig. 6D). We then asked whether DNA replication was reduced in cells overexpressing Polθ-helicase even when those cells were replicating by measuring the fluorescence intensity of EdU in wild-type cells and in cells expressing HA-Polθ-helicase. Overexpression of Polθ-helicase indeed reduced the intensity of the EdU signal (Fig. 6E), while GFP overexpression did not impair the cell growth or EdU incorporation (Fig. 6C–E), showing that DNA replication is not modulated by the overexpression of any protein type. Using CRISPR-Cas9 methodology, we generated a lineage in which Polθ-helicase expression was partially (59%) reduced, and these cells did not exhibit any DNA replication alterations, probably because other factors limit modulation of the replication process (Fig. S2). Together, our data demonstrate that Polθ-helicase modulates DNA replication.

### Overexpression of Polθ-helicase reduces the recruitment of MCM helicase to chromatin

**Figure 7:**
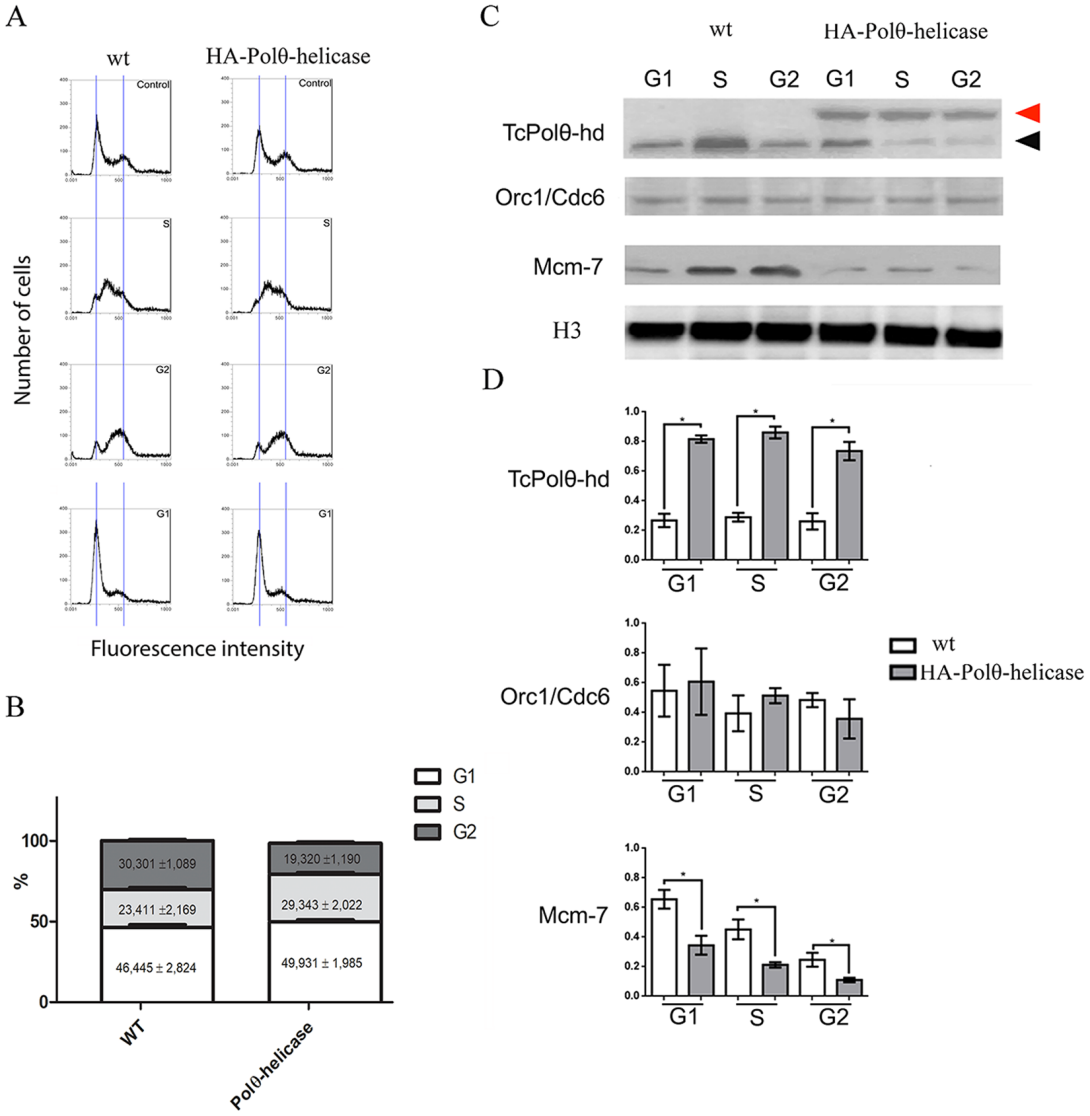
Polθ-helicase overexpression reduces the recruitment of MCM7 to DNA. (**A**) Control cells (WT) and cells overexpressing HA-Polθ-helicase were synchronized with HU. After release, cells were maintained in culture for 6, 18, and 24 h to yield cells at the S, G2 and G1 phases. These samples were stained with propidium iodide and analyzed by flow cytometry according to their DNA content. (**B**) Cell cycle distribution was analyzed by flow cytometry with propidium iodide staining. The fractions of cells in the G1, S and G2 phases were analyzed with AttuneTM NxT software (Life Technologies). Three independent experiments were performed for each analysis presented herein, and statistical analyses were performed using Prism 5 software (GraphPad). (**C**) The same samples obtained in (**A**) were subjected to cell fractionation, during which soluble proteins were discarded, and DNA was treated with DNase to release DNA-bound proteins. DNA-bound proteins were subjected to western blotting using anti-Polθ-helicase, anti-Orc1/Cdc6, anti-MCM7, and anti-histone H3 as the loading control. The black arrow indicates endogenous Polθ-helicase, and the red arrow indicates exogenous Polθ-helicase. (**D**) The bands present in (**C**) were quantified, and the values are expressed as the median and standard deviation of three independent experiments. Quantification of Polθ-helicase is the sum of both endogenous and exogenous Polθ-helicase. Statistical analyses were performed using Student’s t-test, *Indicates p < 0.05.

To confirm the involvement of Polθ-helicase in DNA replication, we analyzed how control and overexpressed cells undergo cell cycle progression. To do this, we synchronized the wild-type and HA-Polθ-helicase lineages with HU and analyzed these cells for different amounts of time after HU release to obtain cells in the G1, S and G2 stages of the cell cycle (Fig. 7A). We verified that in overexpressed cells, entrance into the S phase was delayed (Fig. 7B). The downregulation of nuclear DNA replication after overexpression of Polθ-helicase strongly suggests that Polθ-helicase plays the same role as mammalian Polθ, negatively regulating recruitment of the prereplication component MCM helicase to DNA. To test this possibility, we next analyzed cells in different phases of the cell cycle to assess the presence and amount of Polθ-helicase, Orc1/Cdc6, and MCM7, a subunit of the MCM complex, bound to DNA by extracting DBPs and performing western blot analysis. As expected, high amounts of Polθ-helicase were observed in the G1, S, and G2 phases in cells overexpressing HA-Polθ-helicase. The levels of Orc1/Cdc6 bound to DNA in the G1, S, and G2 phases were similar to those in wild-type cells overexpressing HA-Polθ-helicase. However, cells overexpressing HA-Polθ-helicase could not recruit MCM7 as effectively (Fig. 7C,D), corroborating our hypothesis that Polθ-helicase reduces the recruitment of MCM to DNA in *T. cruzi* compared to that in mammalian cells. In addition, our data showed that the helicase domain of Polθ was sufficient to modulate the recruitment of MCM to DNA.

**Figure 8:**
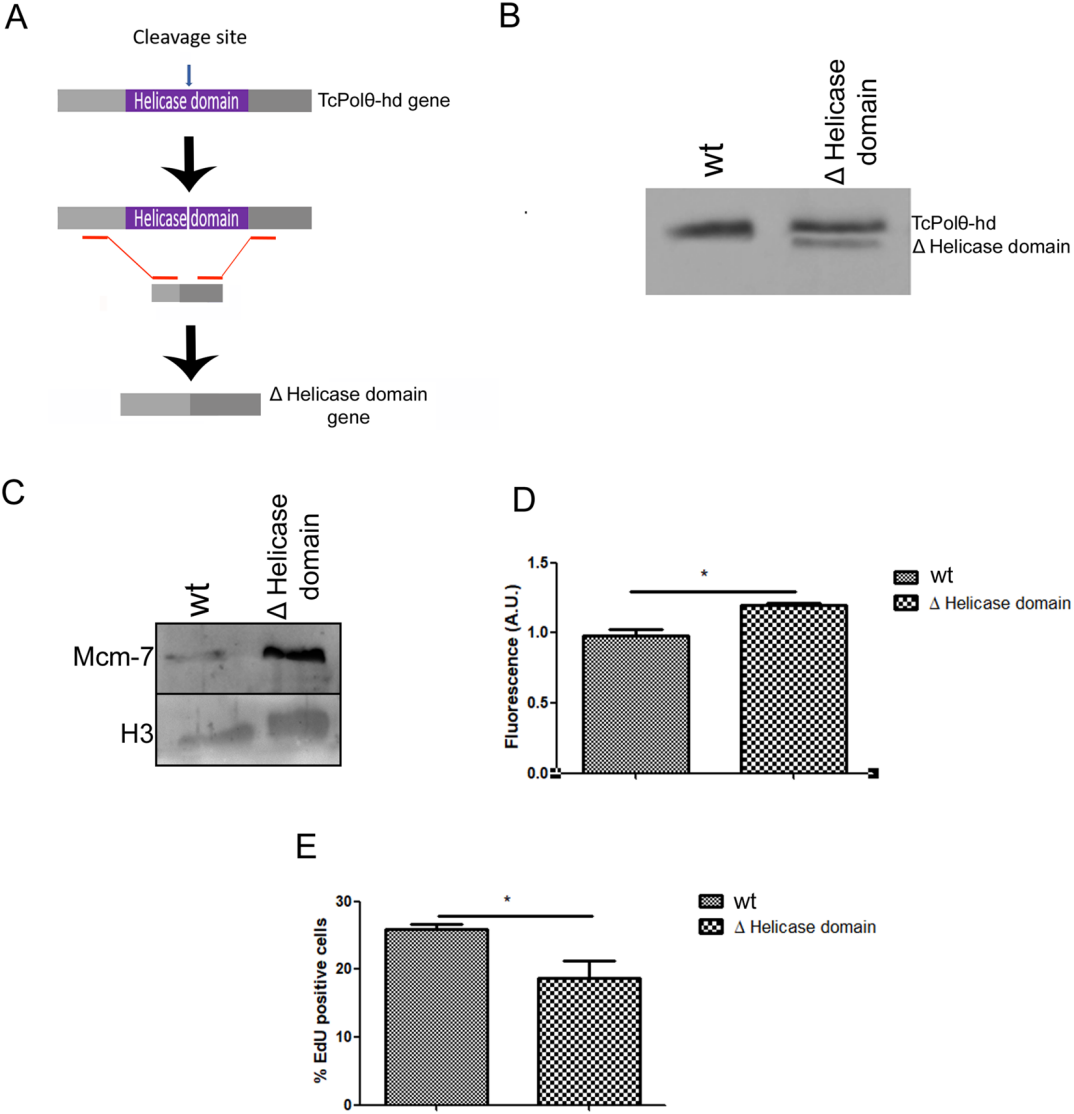
Deletion of Polθ-helicase domain overexpression increases the recruitment of MCM to DNA and impairs DNA replication. (**A**) Schematic representation of deletion of the helicase domain using CRISPR-CAS9 technology. We generated a double-stranded break within the helicase domain and provided a 120 bp fragment containing the 5′ and 3′ regions of the helicase domain as a donor. (**B**) Proteic extracts of wild-type cells and cells from the lineage in which the helicase domain was deleted (Δ Helicase domain) were subjected to western blotting using anti-Tc polθ-helicase antibody. (**C**) Wild-type and Δ Helicase domain cells were submitted to cell fractionation, during which soluble proteins were discarded, and DNA was treated with DNase to release DNA-bound proteins. DNA-bound proteins were subjected to western blotting using anti-MCM7 and anti-histone H3 as the loading control. (**D**) The bands present in (**C**) were quantified, and the values are expressed as the median and standard deviation of three independent experiments. (**E**) The graph shows the percentage of cells that incorporated EdU. Values are expressed as the median and standard deviation of three independent experiments. One hundred cells were analyzed in each replicate.

Because we showed that Polθ-helicase presents helicase activity and that Polθ-helicase modulates the recruitment of MCM to DNA, we next evaluated whether the helicase activity of Polθ-helicase is involved in the recruitment of MCM. To do this, we used CRISPR-CAS9 methodology to generate a lineage expressing a helicase-dead Polθ-helicase. In this assay, we induced cleavage inside the C-terminal helicase domain and provided a donor comprising a 120 bp PCR product containing 5′ and 3′ regions upstream and downstream of the helicase domain, respectively. After cleavage by Cas9, the recombined product obtained using the donor resulted in a new Polθ-helicase lacking the C-terminal helicase domain (Fig. 8A). We confirmed the generation of this mutant (Δhelicase domain) by western blot analysis using anti-Tc Polθ-helicase, as two bands representing wild-type and mutant protein were observed (Fig. 8B). We then extracted DBPs from wild-type and mutant cells and found more MCM7 expression in mutant cells (Fig. 8C,D), strongly suggesting that the helicase activity of Polθ-helicase is involved in the balance of MCM binding to DNA. Finally, we observed reduced DNA replication (visualized by the EdU incorporation assay) in mutant cells (Fig. 8E), supporting data suggesting that the helicase domain is important for the involvement of Polθ-helicase in the DNA replication process. Because mutant cells presented more MCM to DNA, we expected the percentage of EdU incorporated into mutant cells to be similar to than that in wild-type cells. However, the reduction in EdU-positive cells in the mutants showed that helicase activity might be important for DNA replication in addition to its involvement in MCM recruitment.

### HA-Polθ-helicase overexpression impairs origin firing

**Figure 9:**
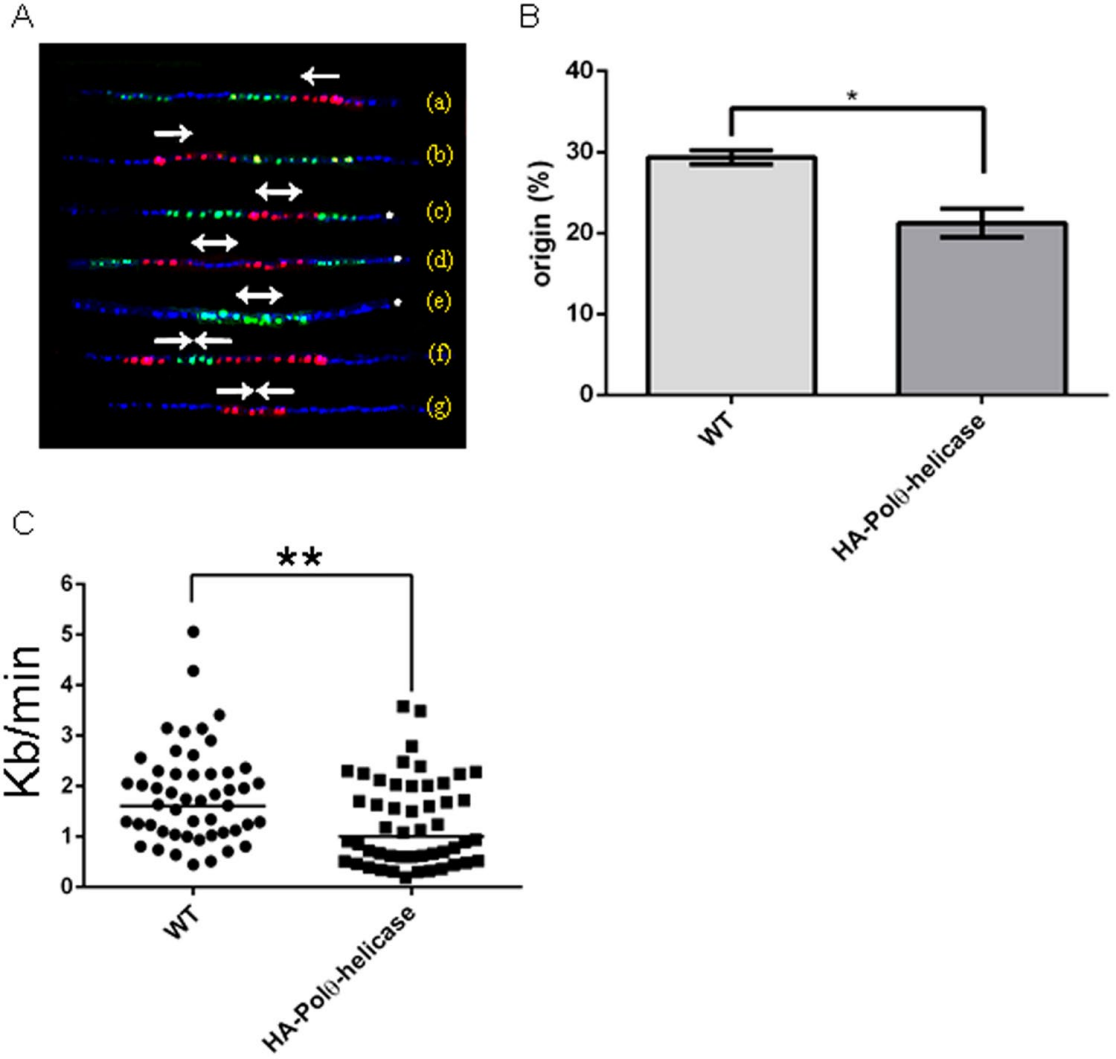
Polθ-helicase overexpression impairs the firing of replication origins. Control (WT) and HA-Polθ-helicase cells were maintained in the presence of IdU (red) and then in the presence of CldU (green). Molecules were stretched onto slides and incubated with anti-DNA (blue) to assess the molecule integrity. Because the fork direction moves from red to green, the fork direction, termination regions and origin regions in molecules can be visualized. (**A**) Patterns found after DNA combing. In molecules (**A**,**B**), only the direction of the fork is observable. Molecules (**C**–**E**) exhibit replication origins, and molecules (**F**,**G**) are termination zones. The arrow indicates the fork direction. *Shows patterns considered to be replication origins. (**B**) The percentage of molecules containing replication origins was determined. Values are the presented as the median and standard deviation of two independent experiments. Fifty molecules were analyzed in each assay. Statistical analyses were performed using Student’s t-test, *Indicates p < 0.05. (**C**) The graph shows the fork speed of each analyzed molecule. The length of the green fragment in kb (between the red and blue signals) was divided by the time of the green pulse in min. Statistical analyses were performed using Student’s t-test, **Indicates p < 0.01.

Finally, we evaluated how reduction in the presentation of MCM to DNA negatively modulated the EdU intensity in cells overexpressing HA-Polθ-helicase. To do this, we performed DNA combing, which allows the visualization of replication origins in replicated DNA molecules stretched onto slides^25^. In this assay, cells were pulsed with the thymidine analog 5-iodo-2-deoxyuridine (IdU) and then pulsed with another thymidine analog, 5-chloro-2-deoxyuridine (CldU). Different primary and secondary antibodies allowed the visualization of each pulse via red (IdU) or green (CldU) fluorescence, while DNA was labeled with anti-ssDNA (blue). Using this approach, we were able to detect the origin and termination regions as well as fork movement. Patterns depicting the regions observed in this experiment are presented in Fig. 9A. We analyzed the frequencies of origins found in control cells and cells overexpressing HA-Polθ-helicase, revealing that 29.38 ± 0.62% of molecules contained origins in control cells, while only 21.25 ± 1.25% of molecules contained origins in cells overexpressing HA-Polθ-helicase (Fig. 9B). We also examined whether alterations in the replication rate could be a factor underlying the downregulation of replication in cells overexpressing HA-Polθ-helicase. To do this, we measured the fork speeds of DNA molecules from control cells and cells overexpressing HA-Polθ-helicase. The speed was obtained by dividing the length of green pulses (in kb) by the duration of a green analog pulse (20 min). We measured the green extension between a red pulse and a DNA signal because we knew that these green fragments were labeled throughout the entire pulse. We found that the fork speed was in fact reduced in cells overexpressing HA-Polθ-helicase. The median fork speed was was 1.829 ± 0.135 kb/min in control cells and 1.285 ± 0.122 kb/min in cells overexpressing HA-Polθ-helicase (Fig. 9C). Representative molecules are presented in Fig. S3. Our data strongly suggest that a reduction in the recruitment of MCM to DNA impairs the firing of replication origins as well as the fork speed.

## Discussion

Mammalian Polθ contains a C-terminal polymerase domain and an N-terminal helicase-like domain, while these domains are split into two different genes in *T. cruzi*. Thus, we herein attempted to answer two main questions. We first tried to determine whether Polθ regulates DNA replication in *T. cruzi* by using Orc1/Cdc6, a component of the *T. cruzi* prereplication complex^16^, as bait in a pull-down assay to search for proteins interacting with the prereplication component. Polθ-helicase was pulled down in this assay, suggesting that this protein interacts with the prereplication complex. Although Polθ-polymerase was not detected in the complex by mass spectrometry, it might still be present because other components of this complex, such as other ORC subunits^17^ and MCMs, were also not detected in this assay. Next, to better investigate the association of Orc1/Cdc6 with Polθ-helicase, we performed an *in vitro* binding assay and observed that Polθ-helicase directly bound to Orc1/Cdc6. Further studies will be important to show when Polθ-helicase interacts with Orc1/Cdc6 during assembly of the prereplication complex. The fact that Polθ-helicase and Orc1/Cdc6 bound to DNA throughout the entire cell cycle suggests that Polθ-helicase may be a constitutive component of the prereplication machinery.

To investigate the possible role of Polθ-helicase in DNA replication, we constructed a cell lineage that overexpressed Polθ-helicase fused to HA via its N-terminal tag (HA-Polθ-helicase). To exclude the possibility that a tag in the Polθ-helicase N-terminal domain would compromise its function, we also generated a cell lineage overexpressing Polθ-helicase fused to HA via its C-terminal domain (Polθ-helicase-HA). In both lineages, the percentage of replicating cells was reduced, and in replicating cells, the incorporation of thymidine analogs was reduced. We then investigated how Polθ-helicase modulates DNA replication, revealing that Polθ-helicase overexpression results in high amounts of Polθ-helicase being bound to DNA and a reduced amount of MCM7 being bound to chromatin. Because the MCM complex is assembled prior to being recruited to DNA (Bell and Dutta, 2002), the reduced amount of MCM7 on DNA might indicate that the amounts of all MCM subunits are reduced in chromatin. A reduction in the recruitment of MCM by Polθ has already been demonstrated in mammalian cells^10^, and we herein showed that overexpression of only Polθ-helicase (lacking the polymerase domain) has the same effect. Like in mammals, the mechanisms involved in the negative modulation of MCM recruitment by Polθ-helicase remain to be elucidated. It is possible that MCM and Polθ-helicase compete for Orc1/Cdc6, although whether MCM and Orc1/Cdc6 are directly associated in *T. cruzi* remains unknown. We observed that cells overexpressing Polθ, which consequently presented lower amounts of MCM to DNA, fired a lower number of replication origins. In fact, accumulating evidence supports the hypothesis that origins with more MCM have more opportunities to fire^26^. Our data also show that a lower amount of MCM on DNA decreases the fork speed. MCM was previously hypothesized to be capable of controlling replisome stabilization via its phosphorylation^27^. Therefore, the lower DNA fork speed observed herein was feasibly a consequence of replisome destabilization caused by less MCM. Additionally, differential replication rates in these two scenarios were previously demonstrated in breast cancer cells overexpressing human Polθ, as control cells showed a median fork speed of 1.699 kb/min, while cells overexpressing human Polθ replicated at a slower speed (1.403 kb/min).

We also demonstrated that *T. cruzi* Polθ-helicase exhibits an ATPase activity that increases in the presence of ssDNA. This DNA-dependent activity has also been observed in Drosophila DNA polymerase θ^19^ and therefore corroborates our conclusion that Polθ-helicase is the metazoan Polθ ortholog. Because our data indicate that Polθ-helicase may be a prereplication machinery component capable of modulating MCM recruitment and DNA replication, the moment at which the cell must rely on Polθ-helicase to control genome duplication can be speculated. The inhibition of origin firing by Polθ-helicase could block DNA replication during an intra-S checkpoint response, when replication might stop allowing damaged DNA be repaired. In mammalian cells, Polθ regulates the timing of DNA replication, changing the activation of early and late origins. However, considering that origin firing is supposedly related to the transcriptional program^28,29^ and that gene expression in *T. cruzi* is mainly controlled at the posttranscriptional level^30^, Polθ-helicase might not be involved in this control in *T. cruzi*. On the other hand, it would be interesting to deeply investigate origin timing control via the Polθ-helicase function in *T. cruzi*, as this would elucidate the relationship (or absence of relationship) between transcription control and origin timing.

Together, our data present Polθ-helicase as a new player in the control of DNA replication in *T. cruzi*. Because some components involved in the licensing of replication origins are very divergent in *Trypanosoma*^15^, the presence of Polθ-helicase in this process as well as that in mammalian cells strongly suggests that Polθ-helicase might be involved in a central control process that has been preserved throughout evolution. The fact that overexpression of only the Polθ-helicase domain modulates DNA replication indicates that if Polθ-polymerase is part of this control, it is not a limiting factor. Nonetheless, the involvement of Polθ-helicase in modulation of DNA replication raises the question of its involvement in genome maintenance and in the genomic plasticity observed in this organism^31^, which are two essential features guaranteeing the success of mammalian host infection.

## Materials and Methods

### Parasites

*T. cruzi* epimastigote forms were cultured in liver infusion tryptose (LIT) medium supplemented with 10% fetal bovine serum at 28 °C. Epimastigotes overexpressing Polθ-helicase were cultured in the same conditions described above in the presence of 500 μg/ml G418 sulfate. HU synchronization was performed as described previously^32^. The synchronization was confirmed by DNA content analysis performed using flow cytometry. Additionally, protein extractions were carried out in each period to analyze and verify the proteins bound to DNA.

### Growth curve

Epimastigotes in the exponential growth phase were harvested, washed in phosphate buffered saline (PBS) (137 mM NaCl, 2.7 mM KCl, 10 mM Na_2_HPO_4_, and 2 mM KH_2_PO_4_, pH 7.4) counted in a Neubauer chamber and distributed in duplicate. Growth was monitored daily by counting parasites in a Neubauer chamber.

### Cell fractionation

Exponentially growing *T. cruzi* epimastigotes (10^8^) were treated with extraction buffer [0.1% Triton X-100, 100 mM NaCl, 10 mM Tris-HCl (pH 7.4), 3 mM MgCl_2_, 300 mM sucrose, 50 mM NaF, 1 mM Na_3_VO_4_, 0.5 mM PMSF, and EDTA-free complete protease inhibitor cocktail (Roche)] at 4 °C for 10 min. Samples were centrifuged (3,800 g x 2 min), and the supernatants were saved (SF1). The pelleted cells were treated with the same extraction buffer and centrifuged (3,800 g x 2 min), and the supernatants were saved (SF2). The pellets were treated with 500 units of DNase I for 30 min and subsequently centrifuged (3,800 g x 2 min), and the supernatants were saved (DBPs). Samples were resolved by SDS-PAGE and analyzed by western blot.

### Epimastigotes overexpressing Polθ-helicase

The gene encoding the *T. cruzi* Polθ-helicase (helicase domain, 2,994 bp, gene ID TcCLB.509769.70, from tritrypdb.org) was amplified from *T. cruzi* Y strain genomic DNA by PCR using the following primers: Polθ_F (**GGGGACAAGTTTGTACAAAAAAGCAGGCTTC** ATGCGGAAGACGTTCGTGTGCC), Polθ_R (**GGGGACCACTTTGTACAAGAAAGCTGG GTC**TGGAAGCGGTGATGCCCGTG) and Polθ_F + Polθ_R_stop **GGGGACCACTTTGTACA AGAAAGCTGGGTC**TTATGGAAGCGGTGATGCCCGTG); the attB1 (primer F) and attB2 (primer R) recombination sites, added to the 5′ end of primers F and R, respectively, are shown in bold. The PCR fragments were inserted into the pDONR221 vector using the BP Clonase enzyme mix from the Gateway recombination cloning system (Thermo Scientific, MA, USA) according to the manufacturer’s protocol. The resulting recombinant plasmids were then used to transfer the Polθ gene (by recombination) into the *T. cruzi* expression vectors pTcGW-3xHA-N and pTcGW-GFP-N [for the cassette containing the PCR-amplified Polθ coding sequence (CDS) with a stop codon (primer Polθ_R_stop)] and into pTcGW-3xHA-C [for the cassette containing the Polθ CDS without a stop codon (primer Polθ_R)] using the Gateway LR clonase enzyme mix, yielding pTcGW-Polθ-3xHA-N, pTcGW-Polθ-3xHA-C and pTcGW-Polθ-GFP-N, respectively. Plasmids pTcGW-3xHA-N and pTcGW-3xHA-C encode three HA epitopes in tandem (3 × HA) for N- and C-terminal tagging, respectively, while plasmid pTcGFP-NH contains the EGFP gene for N-terminal tagging. These expression plasmids are modified versions of the pTcGW 1.1 series Gateway expression vectors constructed for constitutive expression and selection in *T. cruzi*^33^. *T. cruzi* Y strain parasites were transfected with 25 µg of pTcGW-Polθ-3xHA-N, pTcGW-Polθ-3xHA-C or pTcGW-Polθ-GFP-N and selected in LIT medium containing 500 µg ml-1 G418 (Sigma-Aldrich, St. Louis, MO) as previously described^34^. Individual clones from the resistant parasite populations were obtained by cell sorting as previously described^35^.

### Epimastigotes overexpressing GFP

The epimastigote forms were grown to a density of approximately 1 × 10^7^ ml^−1^ cells, harvested by centrifugation at 4,000 x g for 5 min at room temperature, washed once in PBS and resuspended in 0.4 ml of electroporation buffer (140 mM NaCl, 25 mM HEPES, 0.74 mM Na2HPO4, pH 7.5) at a density of 1 × 10^8^ ml^−1^ cells. The cells were then transferred to a cuvette with a 0.2 cm path length, and 50 µg of the pTEX/GFP^36^ plasmid was added. The mixture was placed on ice for 10 min and then subjected to 2 pulses of 450 V and 500 μF with Gene Pulser II (Bio-Rad, Hercules, USA). The electroporated cells were then kept on ice until being transferred into 10 ml of LIT medium supplemented with 10% fetal bovine serum and incubated at 28 °C. After 24 h, 300 μg/ml G418 (Sigma, St. Louis, MO, USA) was added to select transfected parasites, and the transfectants were cloned by serial dilution into 96-well plates.

### rPolθ-helicase and antibody production

The Polθ-helicase (TcCLB.509769.70) CDS was amplified by PCR from *T. cruzi* Y strain genomic DNA and inserted into the pGEM-T easy vector (Promega). Then, the Polθ-helicase CDS was inserted into a pET-28a(+) vector that codes for the 6XHis-tag. The vector was transfected into *E. coli* Bl21 cells, and protein expression was induced using 1 mM isopropyl thio-β-d-galactopyranoside (IPTG) (Fig. S1A). The cells were harvested by centrifugation (3,200 x g, 10 min, 4 °C) and resuspended in lysis buffer (50 mM Tris-HCl (pH 8.0), 50 mM NaCl, 10 mM EDTA pH 8.0 and 1× protease inhibitor cocktail, Roche). The cells were lysed by sonication, centrifuged (18,000 x g, 10 min, 4 °C), and the supernatant was recovered. To obtain purified rPolθ-helicase, the His-tagged protein was purified using Niquel-NTA agarose according to the manufacturer’s instructions (Fig. S1B). rPolθ-helicase was submitted to Proteimax (São Paulo) for the generation of customized specific antibodies.

### Pull-down and mass spectrometry analysis

Exponentially growing *T. cruzi* epimastigotes were lysed with extraction buffer (1.5 M KCl, 20 mM Tris-HCl (pH 7), 3 mM MgCl_2_, 0.5 mM DTT and 1% Tween 20). The epimastigote extract was centrifuged (21,000 g x 15 min) and the supernatant was collected; this proteic extract was used in the subsequent steps as input for pull-down assays. The pull-down assays were performed using His-tagged or rOrc1/Cdc6 MBP (MBP-tagged rPolθ-helicase). The cell lysates were incubated with 100 ng of the tagged protein in a reaction mixture (50 mM Tris-HCl (pH 8), 0.5 mM DTT and 0.1% NP40) overnight at 4 °C. Subsequently, the reaction mixture was incubated with 100 μl of Niquel-NTA agarose (Qiagen) when the His-tagged protein was used or 100 μl of amylose resin high flow when the MBP-tagged protein was used. Then, the samples were centrifuged (230 g for 10 min) and washed several times with wash buffer (50 mM Tris-HCl (pH 8) and 0.5 mM DTT). At the end of the assay, 50% of each pull-down reaction and 10% of the input were fractionated by SDS-PAGE and stained with Coomassie blue. The pull-down reaction fraction (bound fraction) bands were removed from the polyacrylamide gels, stained, and fixed with a solution of 5% acetic acid and 50% methanol for 1 h. Then, the samples were processed for in-gel trypsin digestion and mass spectrometric analysis by LC-MS/MS. Briefly, in-gel digestion was performed according to the method provided by Hanna *et al*. (2000). Each protein band was incubated in a 50% methanol and 5% acetic acid solution, dehydrated in acetonitrile and dried in a SpeedVac. Protein bands in gel plugs were first reduced using 10 mM dithiothreitol for 30 min at 56 °C and then alkylated with 50 mM iodoacetamide at room temperature. Gel plugs were washed with 100 mM ammonium bicarbonate. Then, 50 µg/ml of trypsin (Sigma-Aldrich, St. Louis, MO) was added to the gel plugs and incubated overnight at 37 °C. Digestion was halted with 5% formic acid, and the samples were desalted using Zip Tip C-18 (Millipore) according to the manufacturer’s protocol. Peptides were eluted with 50% acetonitrile/0.1% trifluoracetic acid (TFA).

Mass spectrometry analysis was performed using the LTQ-Orbitrap Velos mass spectrometer (Thermo Scientific) coupled to an EASY-nLC II nanoflow liquid chromatography (Thermo Scientific) with a 35 min gradient of 5 to 95% solvent B (0.1% formic acid in acetonitrile) at a flow rate of 200 nl/min using an in-house prepared precolumn (ID 100 µm x OD 360 µm) packed with 5 cm of C18 10 µm Jupiter beads (Phenomenex, Inc.) attached to an in-house fritted-tip analytical column (ID 75 µm x OD 360 µm) packed with 15 cm of C_18_ 5 µm AQUA beads (Phenomenex, Inc.). Data were acquired in a data-dependent acquisition mode in which the top five precursor ions in each cycle were selected for fragmentation by collision-induced dissociation and excluded for 70 seconds; the nanospray voltage set to 2.3 kV, and the source temperature was set to 250 °C. The ion trap injection time was set to 100 ms, and the FT-MS injection time was set to 100 ms with a resolution of 30,000 across 300–1800 m/z. Raw mass spec data files (.raw) were first converted to Mascot generic format files (.mgf) using MSConvert (ProteoWizard Software Foundation), and MS/MS spectra were searched using Mascot (Matrix Science, version 2.4.0) against a *Trypanosoma cruzi* protein database downloaded from UniProt. The mass tolerance was set to 10 ppm for the precursors and to 0.5 Da for the MS/MS fragment ions. Trypsin was set for enzyme specificity with a maximum of 2 missed cleavages; carbamidomethylation of cysteine was included as the fixed modification. The confidence interval for protein identification was set to 95%, and only peptides with an individual ion score above the identity threshold were considered correctly identified.

### Binding assay

Pull-down assays were performed using the rPolθ-helicase 6× His-tagged and rTcOrc1/Cdc6 MBP-tagged purified recombinant proteins. For each assay, equivalent weights of rPolθ-helicase and rTcOrc1/Cdc6 were used. Both proteins were incubated in a reaction mixture (50 mM Tris-HCl (pH 8), 0.5 mM DTT and 0.1% NP-40) overnight at 4 °C. Subsequently, the reaction was incubated with Niquel-NTA agarose or amylose resin high flow for 4 h at 4 °C. Then, the samples were centrifuged (230 g for 10 min) and washed several times with wash buffer (50 mM Tris-HCl pH 8 and 0.5 mM DTT). At the end of the assay, 50% of each pull-down reaction and 10% of the input were fractionated by SDS-PAGE and subjected to western blot analysis.

### EdU incorporation assays

Exponentially growing epimastigotes were incubated with 100 μM EdU (Click-iT EdU Image Kit, Invitrogen) for 60 minutes. The cells were pelleted, washed with PBS and fixed with 4% (v/v) paraformaldehyde in PBS for 20 min at room temperature. Next, the cells were permeabilized with 0.1% Triton X-100 for 5 min and washed with PBS. Then, the cells were processed using a click chemistry reaction as previously described^37^. The slides were mounted with VECTASHIELD Antifade Mounting Medium and 4′,6-diamidino-2-phenylindole dihydrochloride (DAPI) (Vector lab). Analysis of the number of cells incorporating EdU was carried out by monitoring 200 total cells per coverslip in three independent experiments performed in duplicate using the BX51 microscope (Olympus). The EdU intensity was monitored by quantification of fluorescence intensity, and ImageJ software was used.

### Flow cytometry

*T. cruzi* epimastigotes were sequentially centrifuged (660 × g, 5 min), washed with PBS, and fixed in 70% ethanol overnight at −20 °C. The samples were incubated with propidium iodide (1 mg/ml) and 10 µl of RNase (10 mg/ml) in PBS, and the DNA content was analyzed using the Attune® Acoustic Focusing Cytometer (Applied Biosystems).

### Western blot

Immunoblotting was performed using 10^7^ epimastigotes per lane; the samples were fractionated by 10% SDS-PAGE and transferred to nitrocellulose membranes. The membranes were treated with 5% nonfat dry milk in PBS for 1 h and then incubated with affinity-purified anti-Polθ-helicase (diluted 1:10), anti-TcOrc1/Cdc6 (diluted 1:1,000)^16^, anti-TcMCM-7 (diluted 1:100)^38^, anti-GAPDH (diluted 1:3,000)^39^, and anti-histone H3 (diluted 1:3,000) (Abcam) antibodies overnight at 4 °C. The membranes were washed several times with PBS, and bound antibodies were detected with anti-IgG secondary antibodies coupled to peroxidase (diluted 1:3,000) and a chemiluminescence substrate (Pierce) using standard protocols as described by the manufacturer. Image detection was performed with an UVitec Imaging System (Cambridge). To quantify western blot bands, ImageJ software was used.

### Immunofluorescence

Exponentially growing *T. cruzi* epimastigotes expressing Polθ-helicase-GFP were fixed with 4% paraformaldehyde in PBS for 20 min at room temperature. The samples were permeabilized with 0.1% Triton X-100, blocked with 3% bovine serum albumin (BSA) and incubated with mAbAC, an antibody specific for *T. cruzi* flagellar protein^16^, in PBS 1% BSA for 1 h. Then, the samples were washed with 1× PBS and incubated with an Alexa Fluor 555 secondary antibody (diluted 1:300) (Thermo Scientific). The slides were mounted with VECTASHIELD Antifade Mounting Medium and DAPI (Vector lab) and analyzed under a BX51 microscope (Olympus).

### Helicase activity assay

Helicase activity was measured according to previously established methods^40–42^. The DNA duplex substrate was composed of a pair of oligonucleotides; one was digoxygenin (DIG)-labeled at its 5′ terminus (5′DIG CGATTGGGAGCAGGGTCAGC 3′), and the other was biotinylated at its 5′ terminus (5′biotin GCTGACCCTGCTCCCAATCGTAATCTATAGTGTCACCTA 3′). The dsDNA contained a 3′-tail to initiate unwinding, and the oligonucleotides were annealed. For immobilization of the DNA duplex substrate, each well of a 96-well plate was coated with a 5 μg/ml neutravidin solution, blocked by the addition of a 0.1% (w/v) BSA solution, and incubated at 22 °C for 2 h. Subsequently, the DNA duplex substrate was applied to the 96-well plate, and the mix substrate buffer (PBS containing 1 M NaCl with 2.5 ng of the partially annealed DNA duplex) was added to each well and then incubated at 22 °C for 4 h. Finally, each well was washed with PBS containing 50 mM Tris-HCl (pH 7.5) and 50 mM NaCl. Helicase reactions were initiated upon addition of the reaction mixture [11 nM of purified Polθ-hd, 25 mM 4-morpholine-propanesulphonic acid (MOPS, pH 7.0), 5 mM ATP, 2 mM DTT, 3 mM MnCl_2_, and 100 μg/ml BSA]. For negative controls, the reaction mixture was applied to a well without a DNA duplex substrate. All reactions were carried out for 60 min at 37 °C, and samples were then washed with 150 mM NaCl, dried at room temperature, and washed with detection washing buffer (0.1 M maleic acid, 0.15 M NaCl, 0.3%, Tween 20, pH 7.5). Subsequently, each well was filled with blocking solution (10% BSA (w/v), 0.1 M maleic acid, 0.15 M NaCl, pH 7.5) for 30 min and then incubated in the antibody solution (anti-Dig, Roche, 1:10,000 antibody solution (75 mU/ml) in blocking solution) for 30 min. Next, the wells were washed with detection buffer (0.1 M Tris-HCl, 0.1 M NaCl, pH 9.5), and the chemiluminescence substrate (CSPD – 0.25 mM) was applied to each well; the plates were then incubated at 17 °C for 5 min. The chemiluminescence substrate was removed, the plate was incubated at 37 °C for 30 min, and the chemiluminescence of the remaining DIG label in each well was measured using a luminescence multiwell plate reader.

### ATPase activity assay

ATPase activity was evaluated as described previously^16^ using the Fiske and Subbarow method. The amount of liberated phosphate was quantified based on the calibration line established with Pi standards (0, 2, 6,11 nmol/well). The released inorganic phosphate was quantified by spectrophotometry, and the rate of hydrolysis was determined for each concentration of ATP by linear regression analysis. K_M_ and V_max_ values were determined by fitting the Michaelis-Menten equation to a plot of the hydrolysis rate versus free Pi and analyzed using Prism 5 software (GraphPad).

### TcPolθ knockout and C-terminal helicase domain deletion using CRISPR/Cas9

For knockout (KO) and domain deletion, *T. cruzi* CL Brener epimastigotes expressing Cas9 and T7 RNA polymerase were used; Cas9 was used to generate a locus-directed DSB, and the T7 RNA polymerase was used for the *in vivo* transcription of single guide RNA (sgRNA). KO was performed as described in^43^ with some modifications. For sgRNA *in vivo* transcription, PCR products containing the T7 promoter sequence were used. Two different sgRNAs were designed for the 5′ untranslated region (UTR) (forward primer: 5′GAA ATT AAT ACG ACT CAC TAT AGG gtg ttt ccc act gct cct ctg GTT TTA GAG CTA GAA ATA GC3′) and 3′UTR (forward primer: 5′GAA ATT AAT ACG ACT CAC TAT AGG aag tgc cca gca aag ctg ctG TTT TAG AGC TAG AAA TAG C3′) of TcCLB.509769.70. Forward primers for the 5′ and 3′ UTRs were composed of a T7 promoter sequence (initial capital letters), a 20 bp sequence for Cas9 locus-directed cleavage (lower case letters) and a 20 bp complementary region between these primers and the reverse primers (bold capital letter). Each sgRNA PCR was amplified using the forward primer already mentioned and the same reverse primer (5′AAA AGC ACC GAC TCG GTG CCA CTT TTT CAA GTT GAT AAC GGA CTA GCC TTA TTT TAA CTT GCT ATT TCT AGC TCT AAA AC3′, the letters in bold depict the complementary sequence between the forward and reverse primers). For Donor DNA it was used a PCR product that contains puromycin resistance gene that replaced the TcPolTetha after homologous recombination, which was amplified from pTpuro_v1 plasmid (sequence available at http://www.leishgedit.net/Home.html)^44^ using the following primers: forward: 5′ gtg tgt gtt tgt aat atc taa ttt ctt ttg GTA TAA TGC AGA CCT GCT GC 3′ and reverse: 5′ ttt taa ttg agc gca tcg act gca aga aac CCA ATT TGA GAG ACC TGT GC 3′ (where capital letters are complementary to the plasmid sequences, and lower case letters are the 30 bp used for recombination). A total of 10^8^ epimastigotes were washed and suspended in 0.35 mL of transfection buffer 3 (90 mM sodium phosphate, 5 mM potassium chloride, 0.15 mM calcium chloride, 50 mM HEPES, pH 7.2), which was placed in 0.2 cm cuvettes (Bio-rad) together with 50 µL of TE containing 10 µg of donor DNA and 5 µg of each sgRNA PCR product. The parasites were electroporated using a Bio-Rad gene pulser with two consecutive pulses of 500 µF/450 V each. The parasites were placed into 5 mL of LIT-10% bovine fetal serum and stored at 28 °C overnight; the selective drugs were then added. For domain deletion, sgRNA PCR was amplified using the forward primer 5′ GAA ATT AAT ACG ACT CAC TAT AGG gta tcc ctt ggc tgt acg gag GTT TTA GAG CTA GAA ATA GC 3′ and the reverse primer 5′ AAA AGC ACC GAC TCG GTG CCA CTT TTT CAA GTT GAT AAC GGA CTA GCC TTA TTT TAA CTT GCT ATT TCT AGC TCT AAA AC 3′. The donor DNA used for homologous recombination was a 120 bp PCR product that was amplified from forward (5′ gca gcg ggt ctt ggt aca ccg ccc ttt gtt ttt tcc cag ccg ttg tta gag gaa gaa caa ttt ttg aat cga cgc ggc at 3′) and reverse (5′ ccg cat gag aac ttc atg ctg cca cgg ata cag ttt gat gcc gcg tcg att caa aaa 3′) primers with a 20 bp complementary region (bold letters). The forward primer consisted of 40 bp corresponding to the 5′ region upstream of the domain to be deleted plus 20 bp that were complementary to the reverse primer. The reverse primer was composed of 60 bp corresponding to the 3′ region downstream of the deleted domain. After Cas9 cleavage of the TcPolθ gene (inside the C-terminal helicase domain), the recombination product obtained using the 120 bp PCR product resulted in a new TcPolθ gene sequence lacking the C-terminal helicase domain. After transfection, as described above, the parasites were placed on 5 mL of LIT-10% SFB for 24 h at 28 °C; proteins were then extracted, and genomic DNA was analyzed to evaluate the deletion.

### DNA combing

Exponentially growing epimastigotes (10^8^) were incorporated with two thymidine analogs, IdU- (Sigma-Aldrich, St. Louis, MO) and CldU (Sigma-Aldrich, St. Louis, MO). First, the parasites were incubated with 100 μM IdU for 20 min without an intermediate wash and then incubated with 100 μM CldU for an additional 20 min. After treatment, the cells were embedded in agarose plugs and processed as described previously^25^. DNA molecules were combed on silanized coverslips (Genomic vision) using a DNA combing machine (Genomic vision). The combed DNA was processed using standard protocols as described by the manufacturer (Genomic Vision), and image acquisition was performed using a microscope. For the origin frequency analysis, all patterns, such as fork movement, terminations and origins fired during the first and second pulses, incorporated into a total of 120 molecules for each condition were counted. To determine the significance of the results, Student’s t-test was used. The replication fork speed estimated using intact forks was recorded as ascertained by DNA counterstaining displaying an IdU track flanked by a CldU track. DNA replication parameters generally do not display a Gaussian distribution. Statistical comparisons of the distributions were therefore assessed using the nonparametric Mann–Whitney rank sum test. Two-tailed tests were systematically used. Statistical significance was set at P ≤ 0.05, and a total of 50 molecules were counted for each condition. The analyses were performed in two independent experimental and biological conditions.

### Statistical analysis

Assays for each analysis presented herein were performed in duplicate in three independent experimental and biological conditions, and the data were analyzed using Prism 5 software (GraphPad). Quantitative data are expressed as the mean ± standard error, and the results were statistically analyzed using the Student-Newman-Keuls test. Differences with p values < 0.05 were considered statistically significant.

## Supplementary information

Supplementary Table 1, Figures S1, S2, and S3
