# Supplementary material for: Ortholog of the polymerase theta helicase domain modulates DNA replication in Trypanosoma cruzi
Source: Sci Rep. 2019 Feb 27;9:2888. doi: 10.1038/s41598-019-39348-2 (PMC6393585; doi:10.1038/s41598-019-39348-2)
Supplement: Supplementary file 1 — Supplementary Table 1, Figures S1, S2, and S3 [file 41598_2019_39348_MOESM1_ESM.pdf]

Ortholog of the polymerase theta helicase domain modulates DNA replication in *Trypanosoma cruzi*

Loyze P O Lima[1,2], Simone G Calderano[3], Marcelo S da Silva[1,2], Christiane B Araujo[1,2], Elton J R Vasconcelos[4], Leo K Iwai[5], Claudio A. Pereira[6], Stenio P Fragoso[7], M Carolina Elias[1,2,*]

Supplementary table 1. BLAST analysis parameters

PolQ Helicase domain

Query ID	**Query length (aa)**	**Subject ID**	**Subject length (aa)**	**Species**	**% Query cov**	**% id**	**% sim**	**e-value**
TcCLB.509769.70	998	Tb927.8.3350	1057	*T. brucei*	90	61	74	0,00E+00
TcCLB.509769.70	998	LmjF.23.1380	2242	*L. major*	78	46	57	1,00E-140
TcCLB.509769.70	998	XP_004183803.1	1134	*E. invadens*	78	31	50	5,00E-106
TcCLB.509769.70	998	NP_498250.3	1661	*C. elegans*	72	31	47	3,00E-55
TcCLB.509769.70	998	NP_524333.1	2059	*D. melanogaster*	77	30	50	1,00E-109
TcCLB.509769.70	998	XP_009293682.1	2576	*D. rerio*	75	35	53	3,00E-116
TcCLB.509769.70	998	XP_416549.4	2512	*G. gallus*	72	35	53	1,00E-124
TcCLB.509769.70	998	AAN39838.1	2587	*M. musculus*	75	35	52	5,00E-120
TcCLB.509769.70	998	O75417.2	2590	*H. sapiens*	73	34	51	2,00E-119
TcCLB.509769.70	998	XP_008662598.1 / XP_008662598	2153	*Z. mays*	81	34	51	2,00E-135
TcCLB.509769.70	998	XP_003310356.3	1101	*P. troglodytes*	80	34	55	3,00E-138

PolQ Polymerase domain

Query ID	**Query length (aa)**	**Subject ID**	**Subject length (aa)**	**Species**	**% Query cov**	**% id**	**% sim**	**e-value**
TcCLB.508647.170	881	Tb927.11.5550	846	*T. brucei*	90	38	56	0,00E+00
TcCLB.508647.170	881	LmjF.24.0890	1170	*L. major*	54	36	53	2,00E-94
TcCLB.508647.170	881	XP_004258142.1	591	*E. invadens*	29	32	51	5,00E-27
TcCLB.508647.170	881	NP_498250.3	1661	*C. elegans*	34	29	46	1,00E-23
TcCLB.508647.170	881	NP_524333.1	2059	*D. melanogaster*	35	29	49	3,00E-23
TcCLB.508647.170	881	XP_009293682.1	2576	*D. rerio*	33	28	46	1,00E-25
TcCLB.508647.170	881	XP_416549.4	2512	*G. gallus*	29	28	45	8,00E-21
TcCLB.508647.170	881	AAN39838.1	2587	*M. musculus*	29	31	45	4,00E-24
TcCLB.508647.170	881	O75417.2	2590	*H. sapiens*	29	29	45	6,00E-22

TcCLB.508647.170	881	XP_008662598.1 / XP_008662598	2153	*Z. mays*	34	30	44	2,00E-21
TcCLB.508647.170	881	XP_023445327.1	886	*D. novemcinctus*	34	31	48	3,00E-24
TcCLB.508647.170	881	XP_013968048.1	901	*C. familiaris*	34	31	47	1,00E-23

White and gray subjects are DNA polymerase Theta from different species. Green subjects are HELQ in top table and POLN in the bottom table.

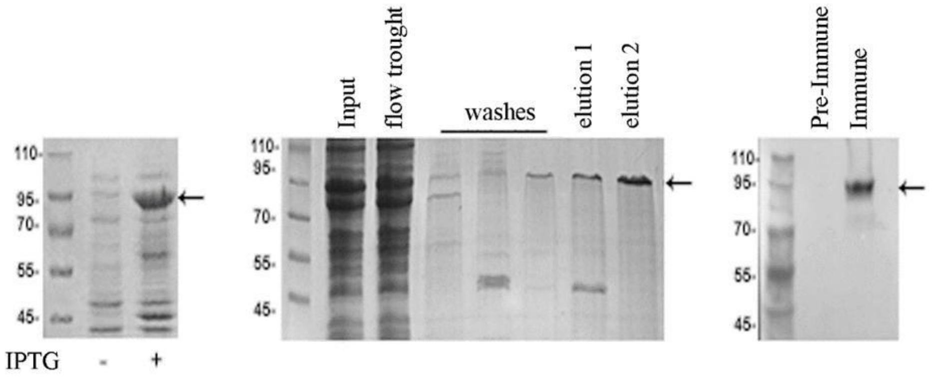

Figure S1. Expression, purification and antibodies generated against rPolθ-helicase. (**A**) rPolθ-helicase was expressed in the presence of IPTG in a prokaryotic system. (**B**) Expressed rPolθ-helicase was purified using a Niquel column. (**C**) Protein extracts from *T. cruzi* epimastigote cells were submitted to SDS-PAGE and transferred onto nitrocellulose membranes that were incubated with anti-rPolθ-helicase (immune) or normal serum (pre-immune) as a negative control.

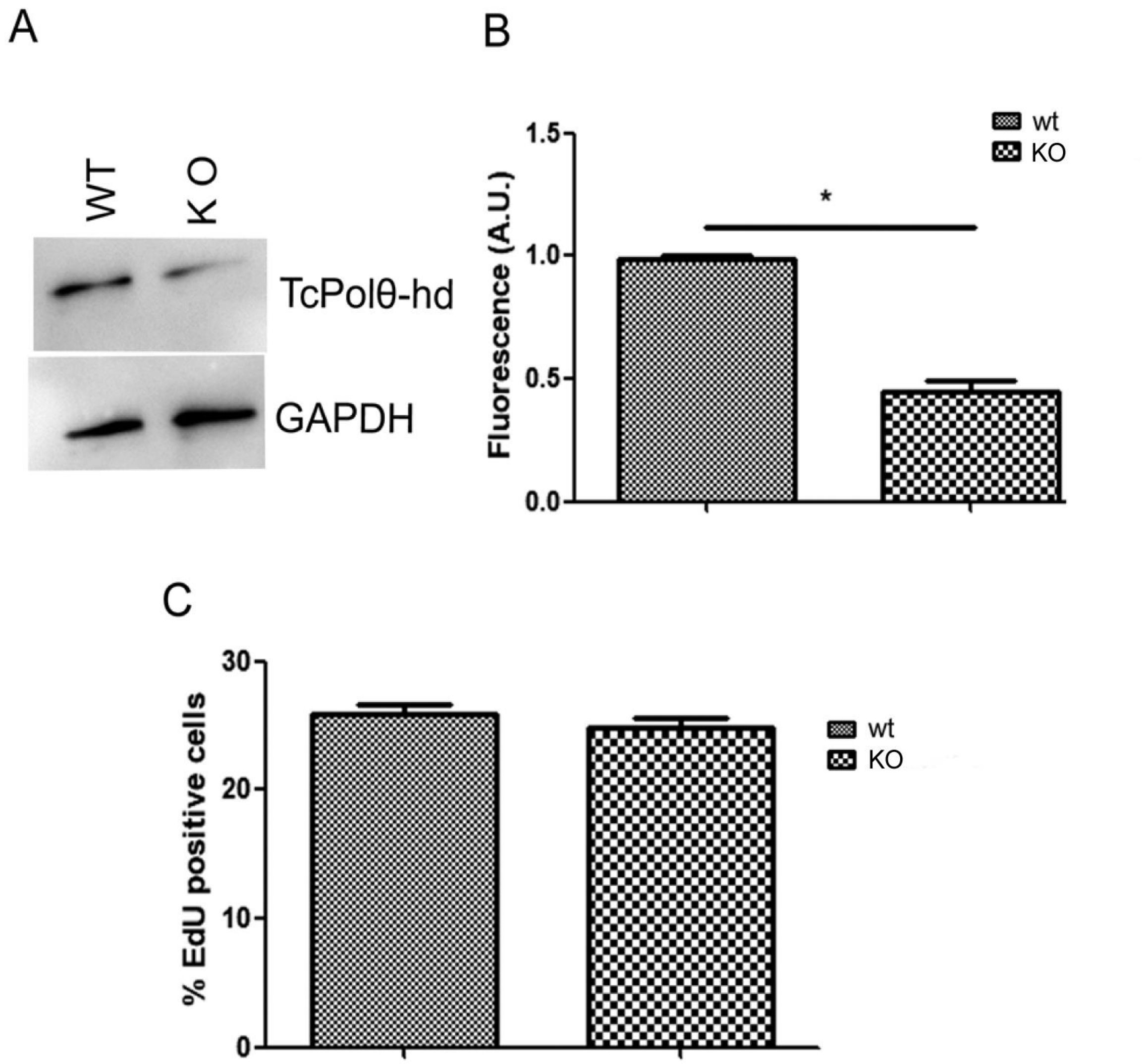

Figure S2. Reduction of rPolθ-helicase expression did not impair EdU incorporation. (**A**) CRISPR-CAS9 methodology was used to generate deletion of Polθ-helicase gene (KO cells). Proteic extracts of control and KO cells were submitted to western blotting using anti- Tc polθ-helicase antibody and anti-GAPDH as loading control. (**B**) Bands present in (**A**) were quantified, and the values are expressed as the median and standard deviation of three independent experiments. (**E**) The graph shows the percentage of cells that incorporated EdU. Values are expressed as the median and standard deviation of three independent experiments. One hundred cells in each replicate were analyzed.

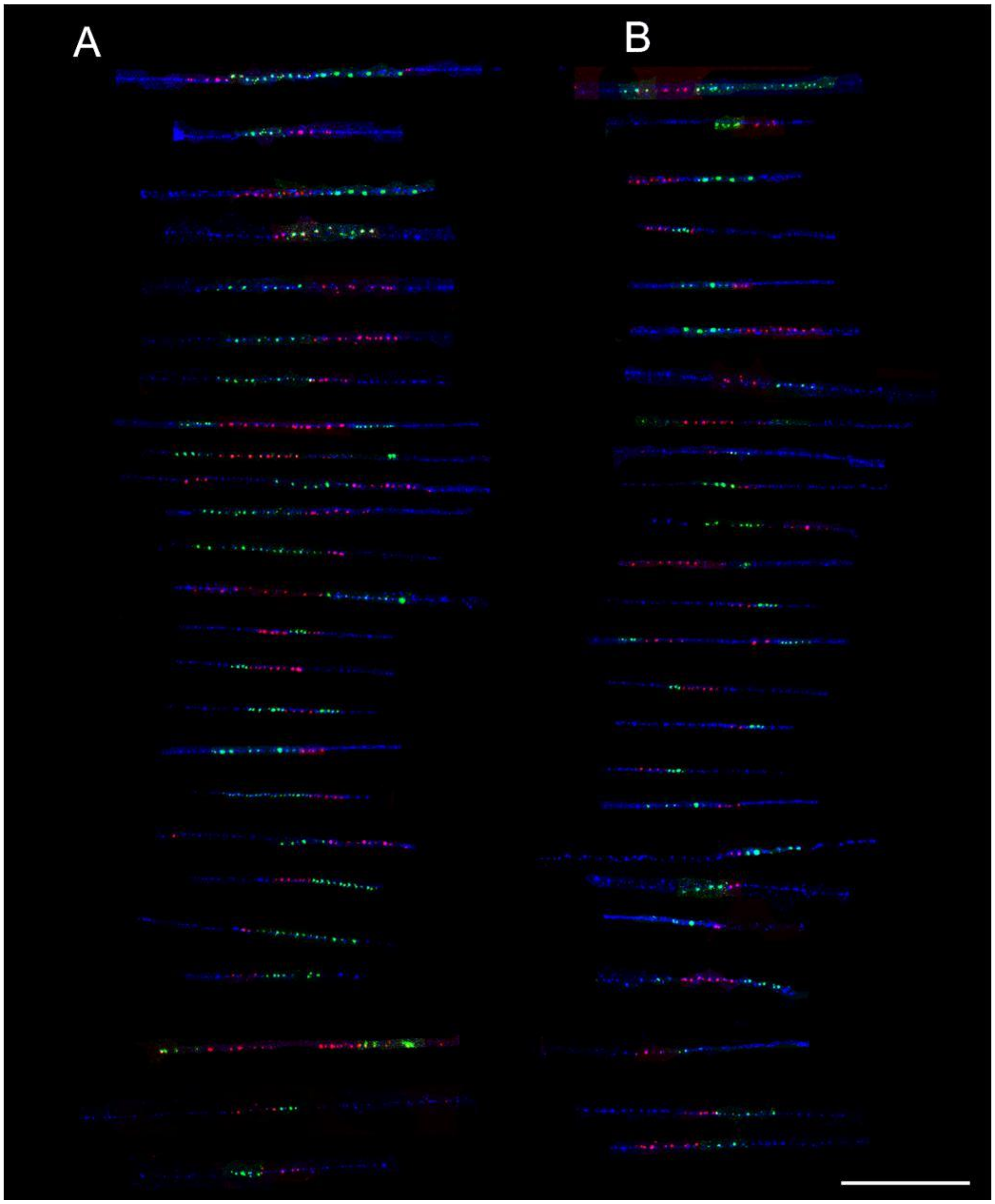

Figure S3. Representative replicated DNA molecules from control and Polθ-helicase overexpressed cells. Control (WT) and HA-Polθ-helicase cells were maintained in the presence of IdU (red) and then in the presence of CldU (green). Molecules were stretched onto slides and incubated with anti-DNA (blue) in order to check the integrity of the molecules. Bar 20 µm.

Uncropped westerns

From Figure 2A

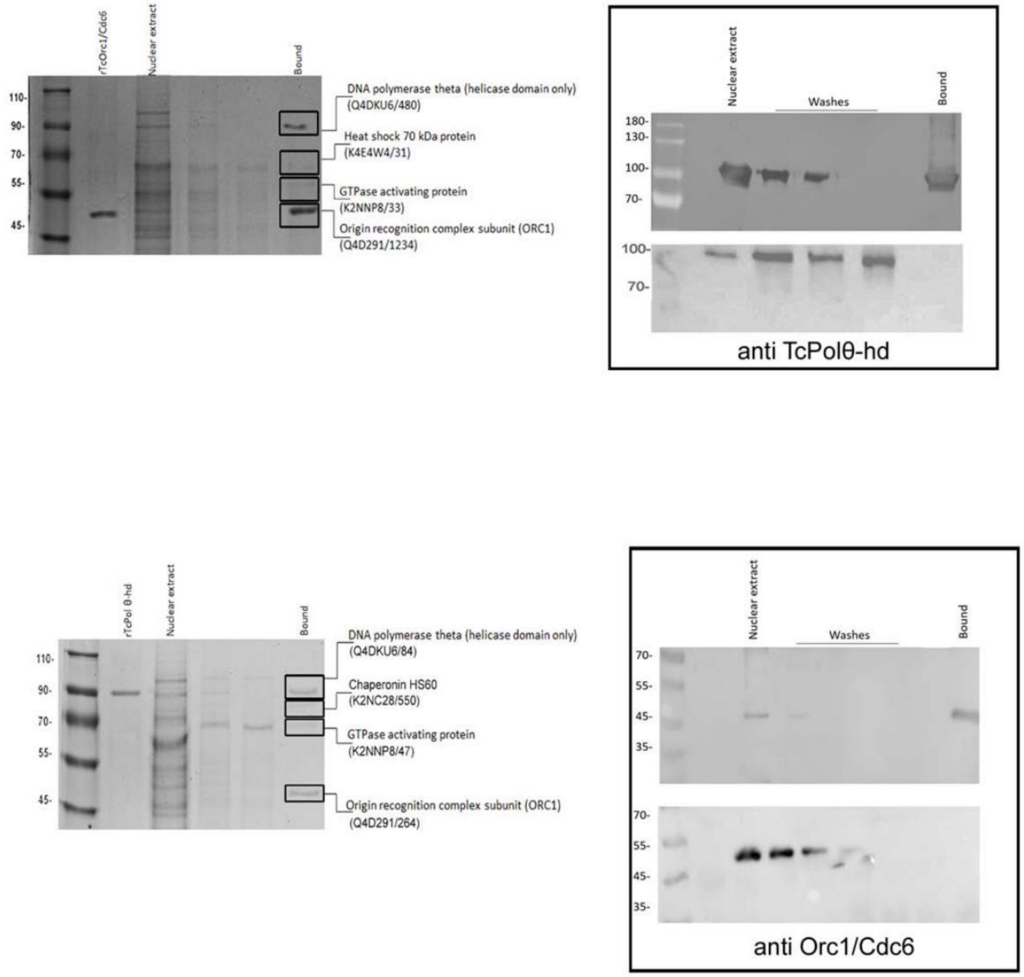

Figure 2. (A) rTcOrc1/Cdc6 (**top panel)** or rPolθ-helicase (**bottom panel**) was used as bait to pull down epimastigote nuclear proteins. Proteins bound to recombinant protein were excised from the gel and analyzed by mass spectrometry. Proteins that also precipitated in the absence of recombinant protein were excluded. Proteins bound to recombinant protein or just to resin were submitted to western blotting analysis using anti- TcPolθ helicase or anti-Orc1/Cdc6 (inserted boxes).

From Figure 2B

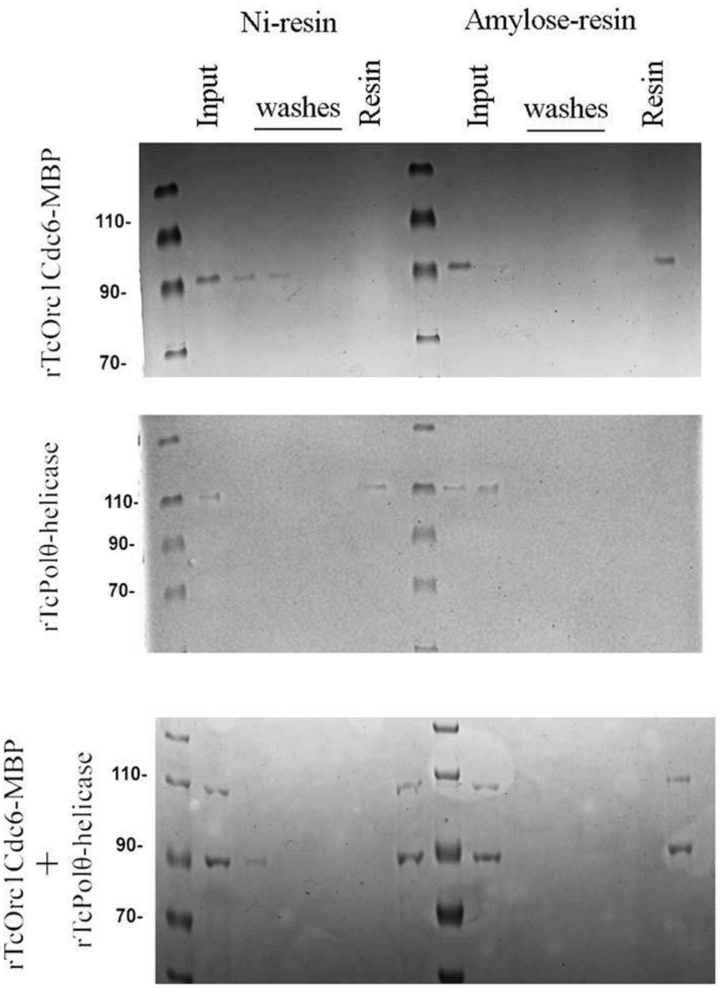

Figure 2. (**B**) rTcOrc1/Cdc6-MBP (**top pannel**), his-rPolθ-helicase **(middle pannel)** or both proteins **(bottom pannel)** were incubated with Ni-resin or with amylose-resin. The Resin lane shows proteins that were captured by each resin.

From Figure 5C

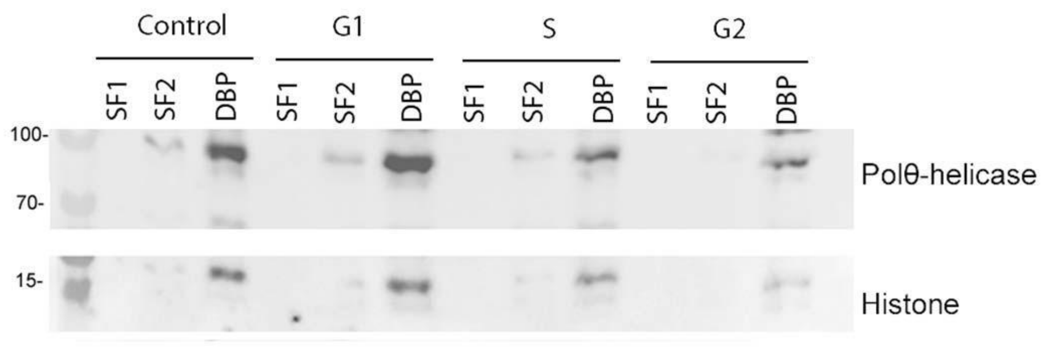

Figure 5 (C) Epimastigote cells were treated with HU for 24 h. Subsequently; cells were washed and maintained in culture for 6 h (S), 18 h (G2), and 24 h (G1). “Control” means cells that were not treated. Samples were submitted to cell fractionation. In this assay, cells were lysed, and after centrifugation, the supernatants were saved as soluble fraction 1 (SF1). Again, pellets were incubated with lysis buffer and centrifuged, and the supernatants were saved as soluble fraction 2 (SF2). Finally, pellets were treated with DNase to obtain DNA-bound proteins and then centrifuged; the supernatants were saved as DNA-bound proteins (DBPs). Samples were submitted to SDS-PAGE and transferred onto nitrocellulose membrane. Membrane was cut and submitted to western blotting using anti-Polθ-helicase and anti-histone H3 (Histone), as a control of the obtainment of DNA-bound proteins in DBP fractions.

From Figure 6A

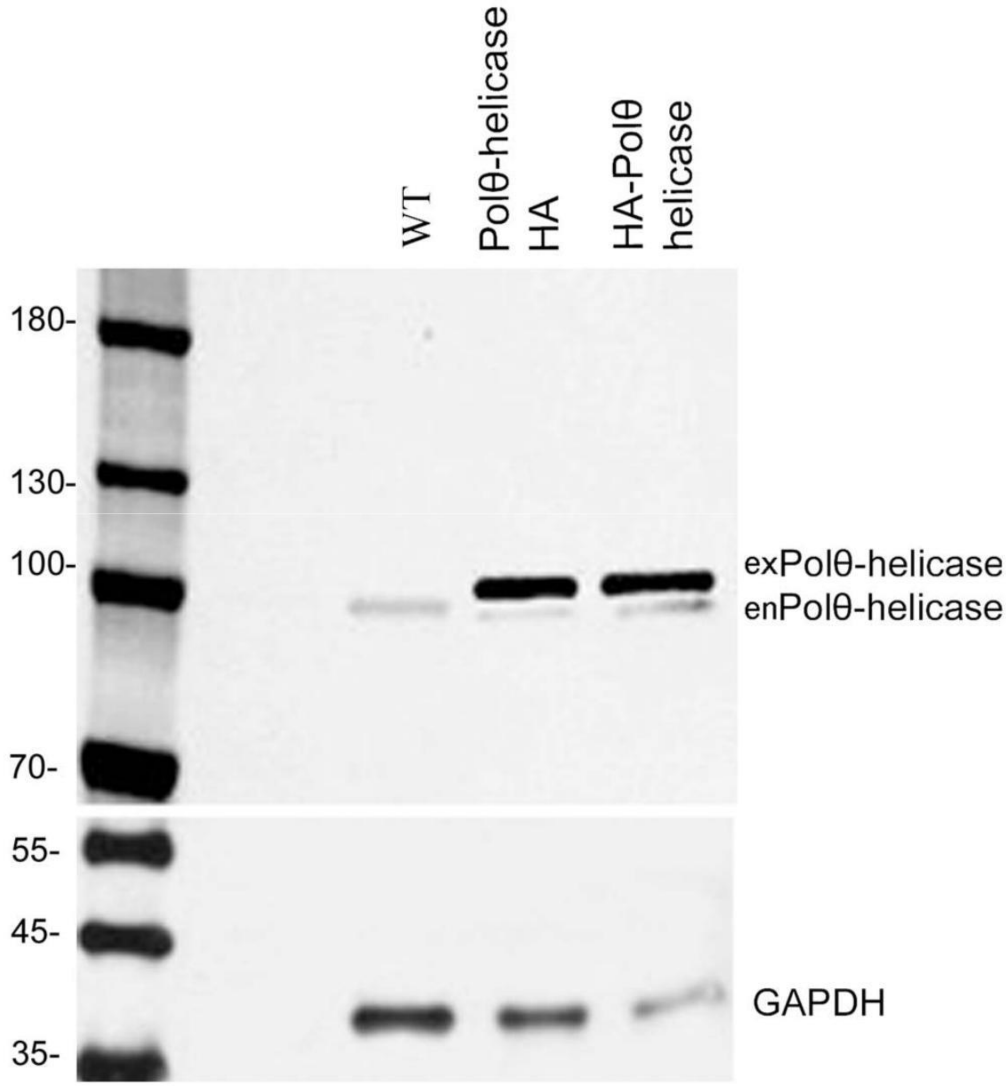

Figure 6. (**A**) Proteic extract of control cells (wt) and cells overexpressing Polθ-helicase fused to HA by its C-terminus (Polθ-helicase-HA) or by its N-terminus (HA- Polθ-helicase) were submitted to western blotting using an anti-Polθ-helicase antibody or anti-GAPDH as the loading control. Endogenous Polθ-helicase is enPolθ-helicase and exogenous protein is exPolθ-helicase.

From Figure 7C

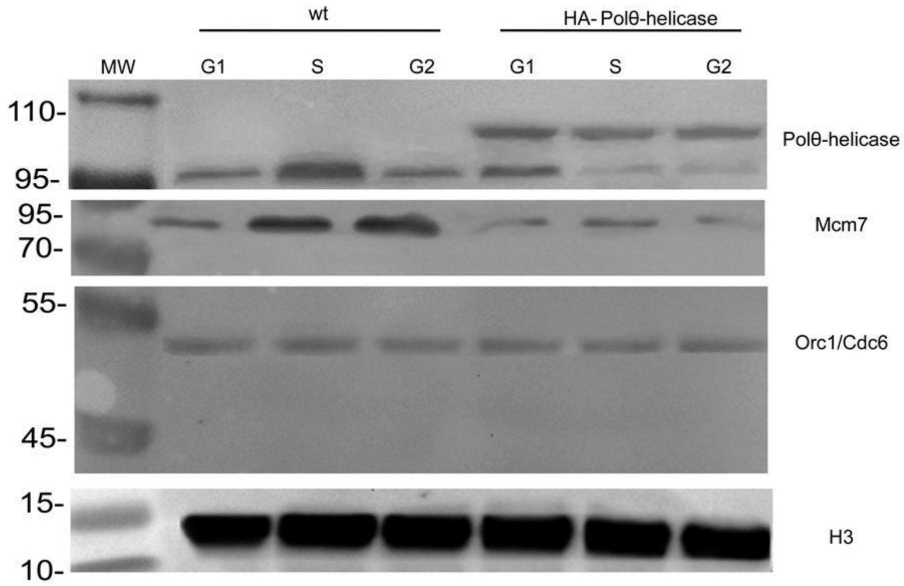

Figure 7. (C) The same samples obtained in (**A – G1, S, G2 from wilt type and HA- Polθ-helicase cells**) were submitted to cell fractionation, where soluble proteins were discarded and DNA was treated with DNase to release DNA-bound proteins. DNA-bound proteins were submitted to SDS-PAGE. Gel was transferred onto nitrocellulose and the mebrane were cut and different pieces were submitted to western blotting using anti-Polθ-helicase, anti-Orc1/Cdc6, anti-Mcm7, and anti-histone H3 as the loading control.

From Figures 8B and C

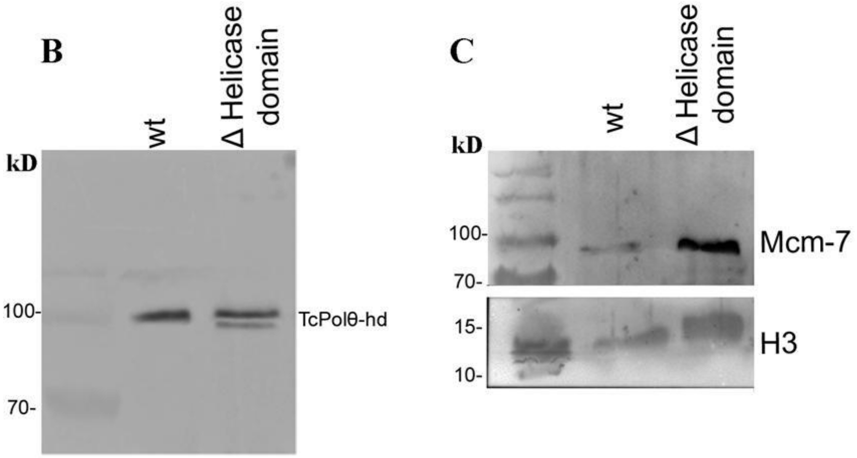

Figure 8. (**B**) Proteic extracts of wild type cells and cells from the lineage presenting deletion of helicase domain (Helicase domain) were submitted to western blotting using anti-Tc polθ-helicase antibody. (**C**) Wild type and Helicase domain cells were submitted to cell fractionation, where soluble proteins were discarded and DNA was treated with DNase to release DNA-bound proteins. DNA-bound proteins were submitted to SDS-PAGE and transferred onto nitrocellulose membrane. Membrane was cut and submitted to western blotting using anti-Mcm7 and anti-histone H3 as the loading control.

From Figure S2A

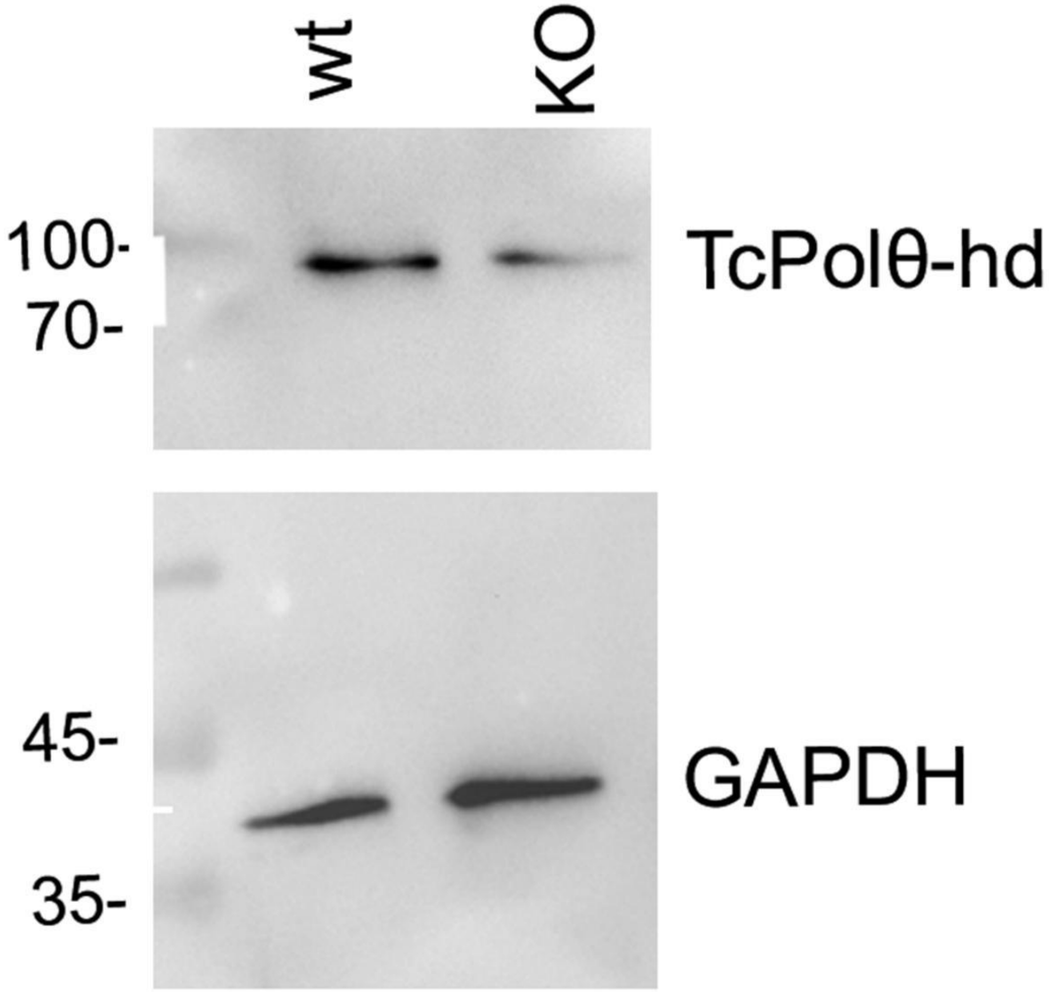

Figure S2. Reduction (**A**) CRISPR-CAS9 methodology was used to generate deletion of Polθ-helicase gene (KO cells). Proteic extracts of control and KO cells were submitted to SDS-PAGE and transferred onto nitrocellulose mebrane. Membrane was cut and submitted to western blotting using anti- Tc polθ-helicase antibody and anti-GAPDH as loading control.
